# Elucidation of the Guanitrypmycin Biosynthetic Pathway in *Kitasatospora azatica* Leading to Structure Revision and Reassignment

**DOI:** 10.1021/acs.jnatprod.6c00242

**Published:** 2026-04-14

**Authors:** Philipp Mann, Shu-Ming Li

**Affiliations:** 9377 Philipps-Universität Marburg, Fachbereich Pharmazie, Institut für Pharmazeutische Biologie und Biotechnologie, Robert-Koch-Straße 4, Marburg 35037, Germany

## Abstract

A recently reported incorrect structure revision and assignment of guanitrypmycins raised our attention and encouraged us to clarify the disagreement with additional experimental data. Genome mining of *Kitasatospora azatica* revealed the presence of a biosynthetic gene cluster for guanitrypmycin-type alkaloids. Expression of this silent cluster in *Streptomyces albus* led to the identification of two *cyclo*-l-Trp-l-Trp (**1**) adducts with guanine (**2a** and **2b**) and two with hypoxanthine (**3a** and **3b**). Detailed NMR analysis and comparison with the published data proved that compound **2a**, which was misassigned to guanitrypmycin C3–1 with an N1–C8′ connection in a previous report, corresponds to guanitrypmycin D3 with a C2–C8′ connection, obtained by a precursor-directed biotransformation. The NMR data of **3b** differ clearly from those of guanitrypmycin C3–4 with a C3–C2′ connection published in 2019 and correspond very well to those of an allegedly revised guanitrypmycin C3–4 with a C3–C8′ connection published in 2024. We therefore corrected this revision and named **3b** guanitrypmycin C3–5.

## Graphical abstract

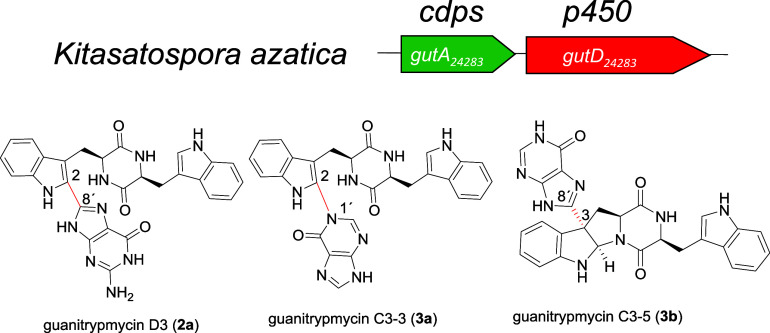

Actinobacteria, especially *Streptomyces* and related genera are renowned for their potential of bioactive natural product production.^1^ Cyclodipeptide (CDP) derivatives, often with a 2,5-diketopiperazine (DKP) skeleton, represent a simple, but very interesting natural product class.^2^ Two different enzyme groups are usually responsible in the formation of a CDP core. While in fungi, bimodular nonribosomal peptide synthetases (NRPSs) are the predominant biocatalysts, bacteria primarily utilize smaller cyclodipeptide synthases (CDPSs).^3,4^ In comparison, NRPSs use free amino acids as substrates under adenosine triphosphate (ATP) consumption for condensation,^5–7^ while CDPSs hijack already activated aminoacyl-tRNAs for the DKP ring formation.^8,9^ CDP core structures contribute significantly to increased stability against proteolysis and also serve as ideal substrates for modification by tailoring enzymes.^10^ Due to their electron-rich indole moiety, tryptophan-containing CDPs are more frequently targeted by such modifications,^11^ e.g., prenylation,^12,13^ methylation,^12^ nucleobase transfer,^14^ dimerization,^15^ and hydroxylation.^16^

Cytochrome P450 enzymes represent one of the most versatile CDPS-associated tailoring enzymes. They catalyze a wide range of modifications, including hydroxylation, aromatization, and complex C–C, C–O or C–N bond formations.^10,17^ In recent years, several biosynthetic gene clusters (BGCs) containing genes for CDPS and P450 enzymes have been identified, including those being responsible for the formation of guanitrypmycins by coupling of a tryptophan-containing DKP with a nucleobase like guanine or hypoxanthine via different C–C or C–N bonds (Figure 1).^14,18–22^ With the growing number of described guanitrypmycins, misassignment or incorrect structural revision has been reported in the literature. For instance, guanitrypmycin D3 obtained from a precursor-directed biosynthesis experiment in 2023,^21^ was later misassigned to guanitrypmycin C3–1.^14^ Guanitrypmycin C3–4 with a C3–C2′ connection published in 2019 ^19^ was incorrectly revised to a structure with a C3–C8′ connection.^14^

**Figure 1:**
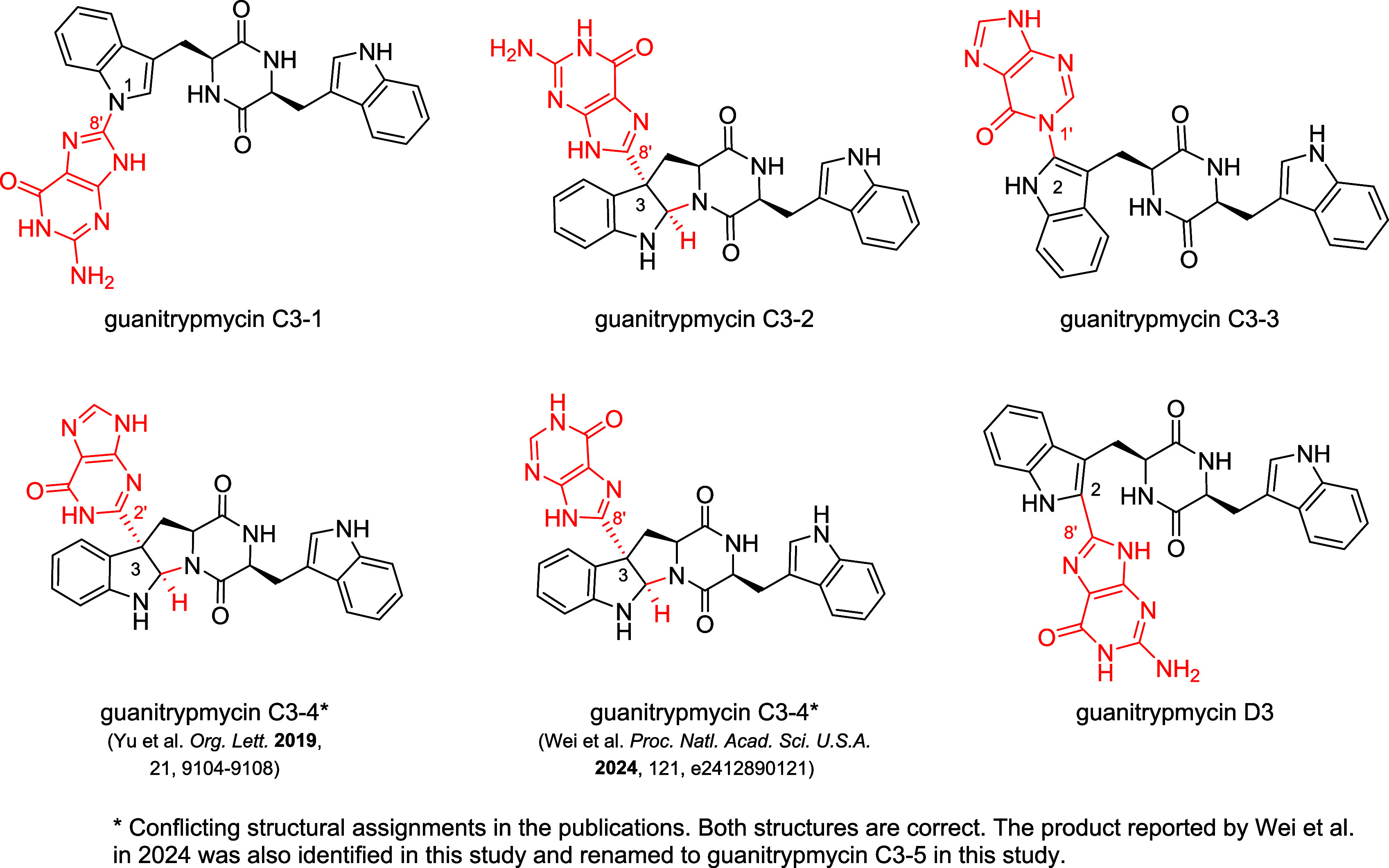
Structures of representative guanitrypmycins with a cWW (*cyclo*-l-Trp-l-Trp) scaffold described to date.

In this study, we investigated the genomic potential of *Kitasatospora azatica* (syn. *Streptomyces azaticus*) NRRL B-24283 (= *Kitasatospora azatica* KCTC 9699). Genome mining revealed the presence of a promising guanitrypmycin-like BGC. Therefore, a heterologous expression strategy in *Streptomyces albus* was accessed to investigate the chemical diversity encoded by this BGC. Herein, we report the successful activation of the cluster, leading to identification of three guanitrypmycins. After structural elucidation, we confirmed the misassigned guanitrypmycin C3–1 is guanitrypmycin D3. We also corrected the revised structure of guanitrypmycin C3–4 back to a C3–C2′ connection between *cyclo*-l-Trp-l-Trp (cWW, **1**) and hypoxanthine and termed the product with a C3–C8′ connection guanitrypmycin C3–5.

## Results and Discussion

### Comparison of NMR Data of the Controverse Guanitrypmycin Structures

In a previous study, two BGCs *gut*_2774_ and *gut*_5414_ coding for a CDPS and a cytochrome P450 enzyme were identified in the genomes of *Streptomyces lavendulae* NRRL B-2774 and *Streptomyces xanthophaeus* NRRL B-5414, respectively.^19^ Both clusters are responsible for the formation of four guanitrypmycins including guanitrypmycin C3–4 (Figure 1).^19^ Wei et al. reported in 2024 the isolation of five metabolites after feeding cWW into a *Mycobacterium smegmatis* strain harboring *ptmB* from *Kitasatospora mediocidica*, coding for a cytochrome P450 enzyme.^14^ They identified two products coupled with guanine and two with hypoxanthine. One of the products was assigned to guanitrypmycin C3–1 and another to guanitrypmycin C3–4 with a proposed structure revision from the original C3–C2′ to C3–C8′ connection (Figure 1).^14^ However, a detailed reevaluation of the ^1^H and ^13^C NMR data reported for these two compounds (all recorded in DMSO-*d6*)^14^ revealed clear discrepancies with those published by our group before,^18,19^ especially for signals near the nucleobase moieties. The NMR data of guanitrypmycin C3–1 reported by Wei et al.^14^ correspond to guanitrypmycin D3 reported by Liu et al.^21^ (see below for detailed comparison of characteristic chemical shifts). These significant data deviations strongly indicated an incorrect structure revision or misassignment in the publication by Wei et al.^14^

### Identification of the *gut*_24283_ Gene Cluster

To address this issue, we initiated a genome-mining strategy to identify a BGC with high homology to that in *Kitasatospora mediocidica* and found one candidate in the genome of *Kitasatospora azatica* KCTC 9699 (= NRRL B-24283). The CDPS GutA_24283_ (247 aa, accession number WP_083976074.1) encoded by *gutA*_24283_ and the cytochrome P450 enzyme GutD_24283_ (398 aa, WP_035841180.1) encoded by *gutD*_24283_ share high sequence identities of 72.6 and 86.7% with PtmA and PtmB, respectively (Figure 2).

**Figure 2:**
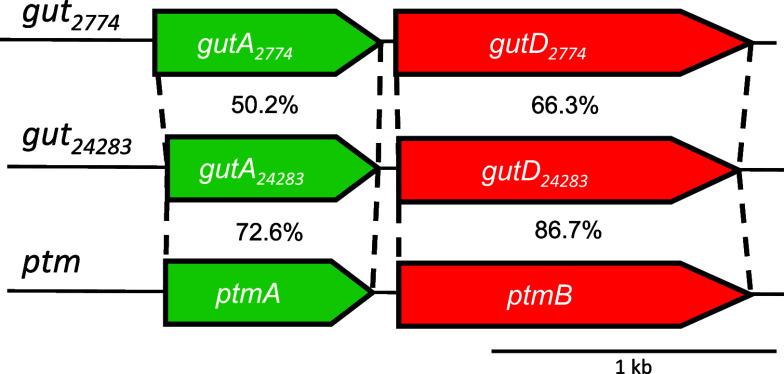
Putative *gut*_24283_ cluster from *K. azatica* compared with the homologous clusters from *S. lavendulae* (*gut*_2774_) and *K. mediocidica* (*ptm*).

The remarkable sequence identities, especially between the two cytochrome P450 enzymes in *K. azatica* and *K. mediocidica* (Figure 2 and Table S1), suggest similar or even same products of these BGCs. This scenario offers an opportunity to reevaluate the chemical identity of their metabolites.

### Heterologous Expression of the *gut*_24283_ Cluster

The *gut*_24283_ cluster seems to be silent under standard laboratory conditions, which was confirmed by liquid chromatography–mass spectrometry (LC-MS) analysis of an EtOAc extract of *K. azatica* culture in glucose–yeast–malt (GYM) *Streptomyces* medium (Figure S1). Therefore, we initiated a heterologous expression in *Streptomyces albus* J1074 and focused first on the standalone activity of *gutA*_24283_. The gene of interest was amplified from *K. azatica* NRRL B-24283 and cloned into the replicative *Streptomyces* expression vector pPWW50A via homologous recombination in *E. coli* DH5α to generate the construct pPhM02.^23^ Introduction of pPhM02 into *Streptomyces albus* was achieved by conjugation using *E. coli* ET12567.^24^ The pPhM02-carrying *Streptomyces albus* strain was cultivated at 28 °C in modified R5 medium for 7 days. LC-MS analysis of the EtOAc extract revealed an additional peak with a [M + H]^+^ ion at *m*/*z* 373.1659 compared to the negative control strain carrying the empty vector pPWW50A (Figure 3).

**Figure 3:**
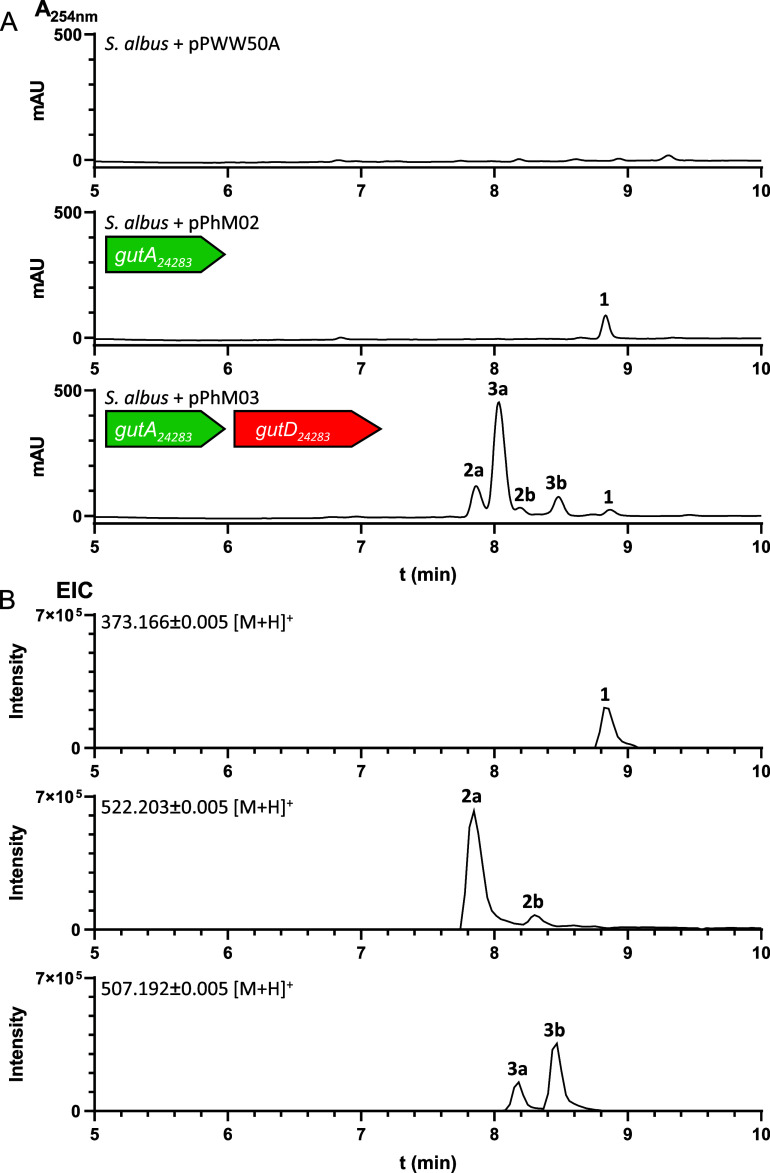
LC-MS chromatograms of the culture extracts of *S. albus* conjugants harboring different constructs after 7d cultivation in modified R5 medium. (A) UV absorptions for 254 nm are illustrated. (B) Extracted ion chromatograms (EICs) of [M + H]^+^ ions of the relevant compounds.

Isolation and NMR analysis confirmed this metabolite to be cWW (**1**) and proved GutA_24283_ as a functional cWW synthase. Subsequently, the construct pPhM03 containing *gutA*_24283_ and *gutD*_24283_ was expressed in *S. albus*. LC-MS analysis of the extract showed five new peaks, which are absent in the control strain (Figure 3). In addition to **1**, two of them (**2a** and **2b**) are likely coupling products of cWW and guanine with [M + H]^+^ ions at *m*/*z* 522.2026. The other two (**3a** and **3b**) with [M + H]^+^ ions at *m*/*z* 507.1920 could be cWW-hypoxanthine adducts. This indicates that the P450-enzyme functions as a nucleobase transferase.

### Structural Elucidation of the *gut*_24283_ Cluster Products

To determine the structures, products **2a**, **3a** and **3b** were isolated from large-scale fermentation extracts and characterized by MS (Figure S2) and NMR analyses (Figures S6–18). Due to low quantity, the structure of **2b** could not be elucidated in this study. Compound **3a** was identified as guanitrypmycin C3–3 by comparison of its ^1^H NMR data (Table S4 and Figure S12) with those published previously.^19^ Guanitrypmycin C3–3, featuring a hypoxanthine moiety linked via its N1′ to C2 of cWW, was also found in both *ptm* and *gut*_2774_ clusters.^14,19^

A visual comparison reveals that the ^1^H NMR spectrum of compound **2a** perfectly matches the spectrum of the alleged guanitrypmycin C3–1 provided by Wei et al. in the Supporting Information.^14^ However, as illustrated in Figure 4, the reference spectrum of authentic guanitrypmycin C3–1 published by our group in 2018 ^18^ differs clearly from that of **2a**, especially regarding the signals for NH-1, NH-1′, H-2, H-7, and NH-20. Instead, the ^1^H and ^13^C NMR spectra of **2a** perfectly aligns with that of guanitrypmycin D3 (Figures 4 and S3), which was previously isolated after feeding cWW to a heterologous *S. albus* strain carrying the cytochrome P450 gene *gutD*_1521_ from *Streptomyces* sp. NRRL S-1521.^21^ Isolation of **2a** from the *S. albus* strain expressing the *gut*_24283_ cluster confirms its *de novo* generation. This unequivocally proves that the product isolated by Wei et al. is in fact guanitrypmycin D3 rather than guanitrypmycin C3–1.

**Figure 4:**
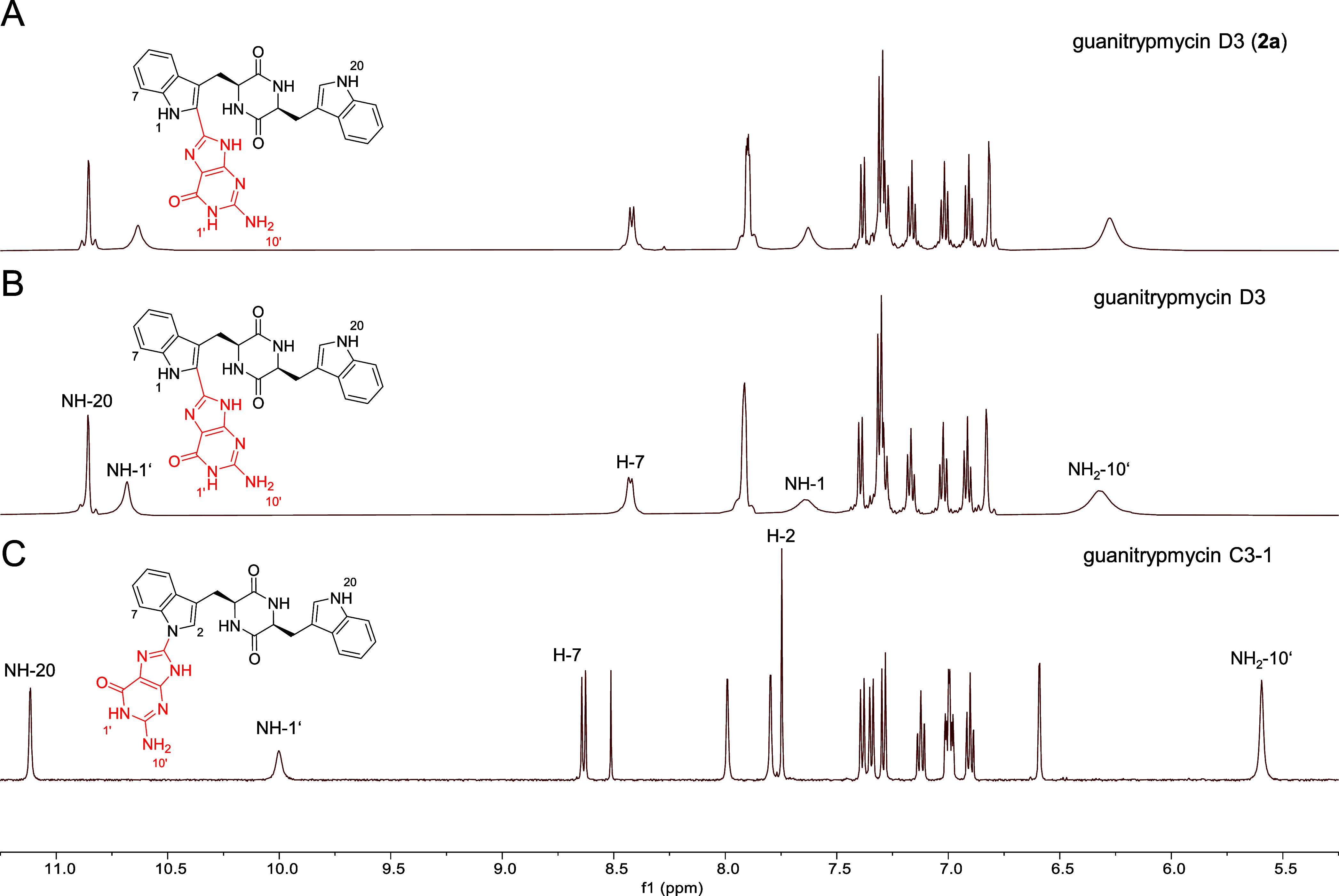
Comparison of ^1^H NMR spectrum of **2a** (DMSO-*d*_6_) with those of guanitrypmycin C3–1 and D3 in the range of 5.25–11.25 ppm. Characteristic signals for the two structures are labeled. (A) Spectrum of compound **2a** isolated in this study, identified as guanitrypmycin D3. (B) Spectrum of guanitrypmycin D3. The spectrum was generated using raw data originally reported by our group.^21^ (C) Spectrum of guanitrypmycin C3–1. The spectrum was also generated using raw data originally reported by our group.^18^

The structure of compound **3b** was elucidated by extensive interpretation of its NMR data (Table S5), including ^1^H NMR, ^13^C NMR, ^1^H–^1^H COSY, HSQC, HMBC, and NOESY spectra (Figures S13–18). We observed that the NMR data of **3b** perfectly match those reported by Wei et al.^14^ for their “revised” version of guanitrypmycin C3–4. However, a direct comparison reveals that the ^1^H and ^13^C NMR spectra of **3b** differ clearly from those of our original guanitrypmycin C3–4 published in 2019 (Figures 5 and S4),^19^ e.g., the absence of the signal for H-8′ and the presence of the signal for H-2′ as well as the deviation of the signal for NH-1 (Figure 5).

**Figure 5:**
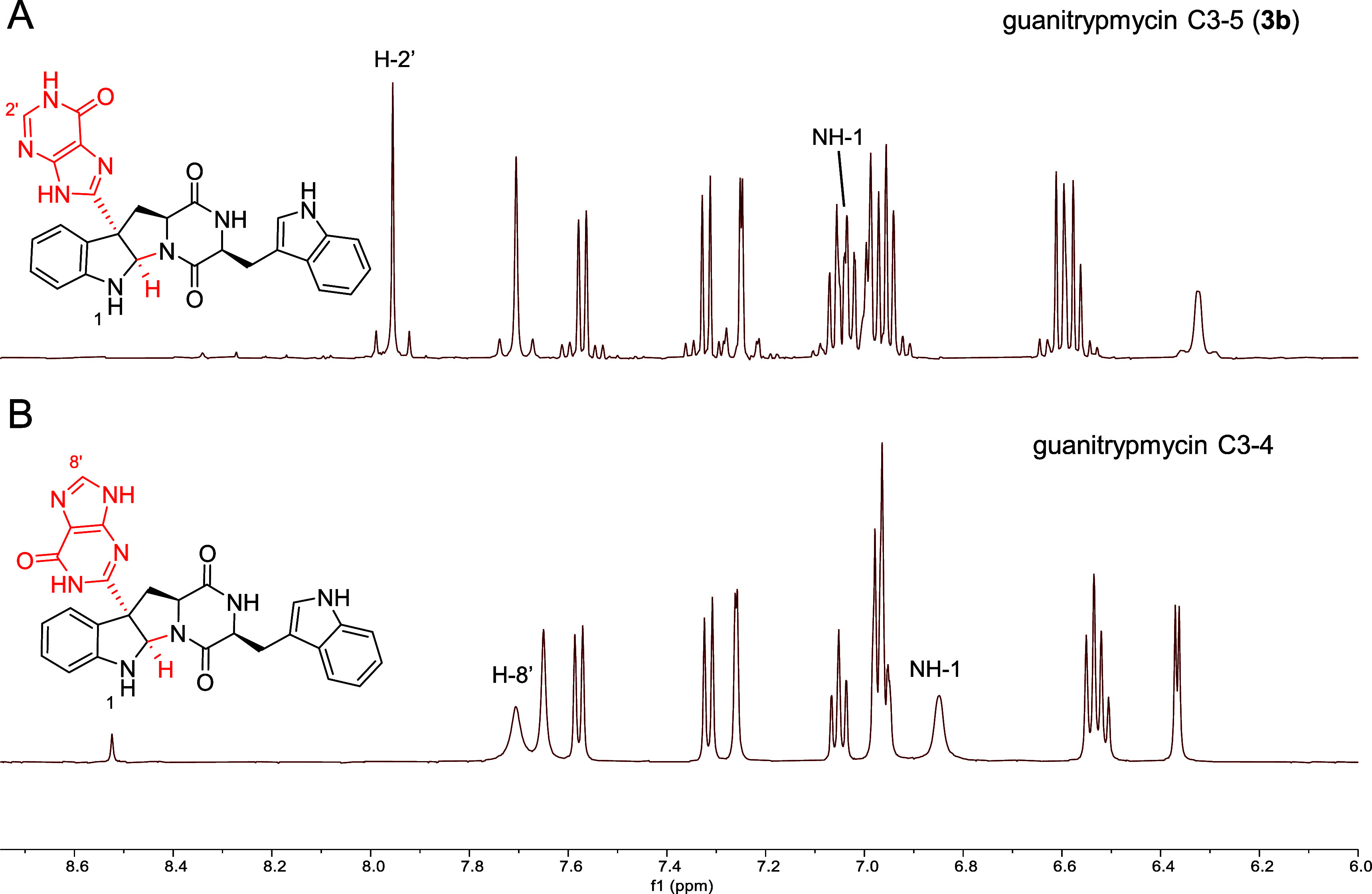
Comparison of ^1^H NMR spectrum of **3b** (DMSO-*d*_6_) with that of guanitrypmycin C3–4 in the range of 6.0–8.75 ppm. Characteristic signals of both structures are labeled. (A) Spectrum of compound **3b** isolated in this study, identified as guanitrypmycin C3–5. (B) Spectrum of guanitrypmycin C3–4. The spectrum was generated using raw data originally reported by our group.^19^

This unambiguously confirms that the compound produced by PtmB and GutD_24283_ (**3b**) represents a new guanitrypmycin, structurally distinct from the original GutD_2774_ product guanitrypmycin C3–4. Consequently, Wei et al. successfully elucidated the structure of the isolated metabolite, but incorrectly proposed it as a revision of C3–4.^14^ We therefore reject the structure revision by Wei et al.,^14^ reestablish the original structure of guanitrypmycin C3–4 as published by Yu et al. in 2019,^19^ and name **3b** guanitrypmycin C3–5. Our reassignment and correction for those structures in the publication of Wei et al.^14^ are also plausible in the context of the high sequence identity between the two cytochrome P450 enzymes PtmB and GutD_24283_.

In conclusion, heterologous expression of the *gut*_24283_ cluster from *K. azatica* successfully unlocked the chemical diversity encoded by this biosynthetic pathway. It can be proposed that the CDPS GutA_24283_ uses two molecules of Trp-tRNA^Trp^ to assemble cWW (**1**), which is then modified by transfer of a guanine and hypoxanthine with the cytochrome P450 enzyme GutD_24283_, leading to the formation of four coupling products (**2a**, **2b**, **3a** and **3b**, Scheme 1). Elucidation of structures of **2a** and **3b** allowed us to reassign or to correct the structure revision made by Wei et al. in 2024.^14^

**Scheme 1:**
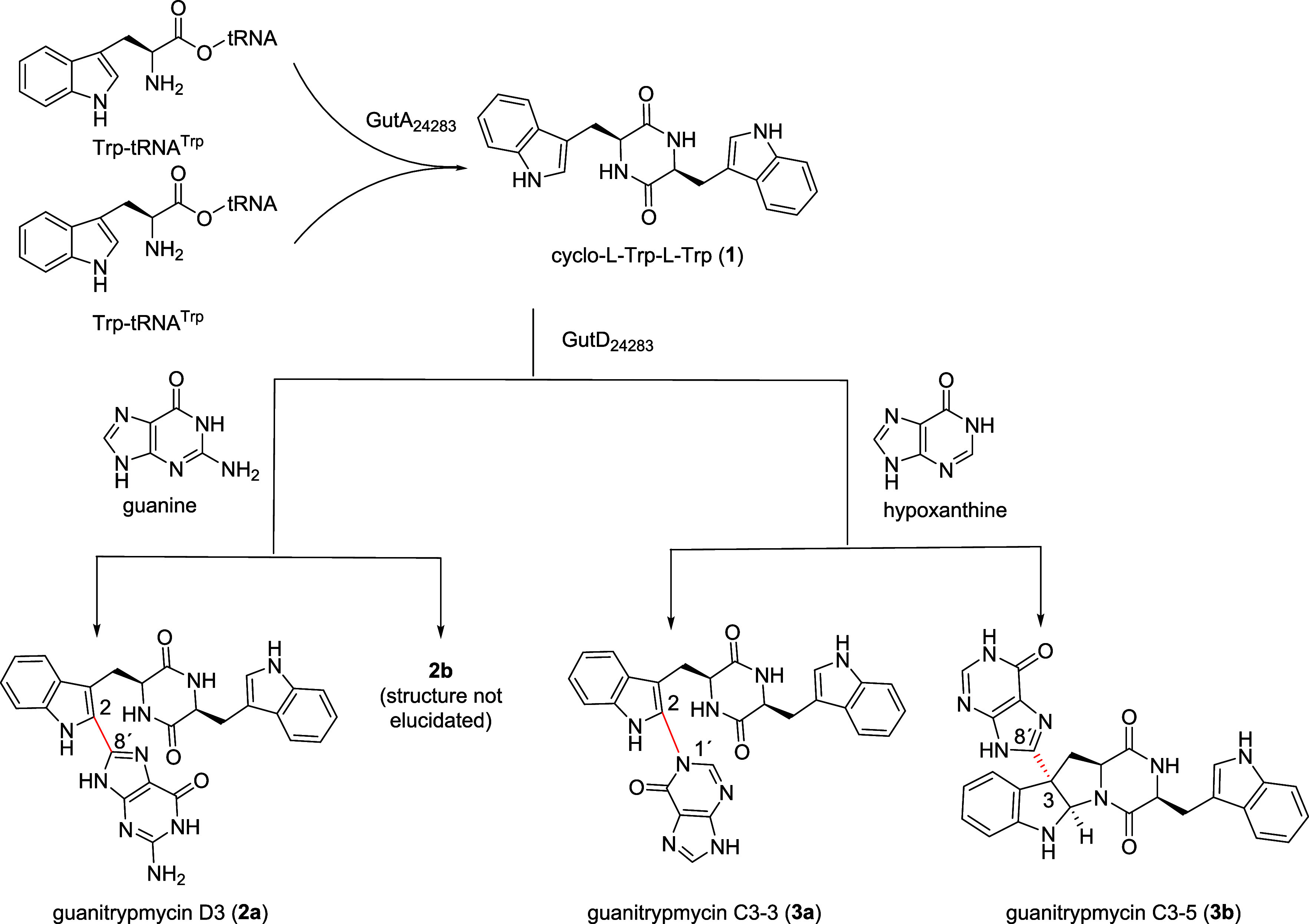
Proposed Biosynthetic Pathways for the Formation and Metabolism of cWW by Gut_24283_ Enzymes

## Experimental Section

### General Experimental Procedures

NMR spectra were recorded at room temperature on a JEOL ECA-500 spectrometer (JEOL, Tokyo, Japan). All samples were dissolved in DMSO-*d*_6_. Chemical shifts are referenced to those of the solvent signals. Product isolation on a semipreparative HPLC was performed on an Agilent HPLC series 1200 (Agilent Technologies, Böblingen, Germany) equipped with a VDSpher PUR 100 C_18_-M-SE column (250 × 10 mm, 5 μm) (VDS Optilab Chromatographie Technik, Berlin, Germany). Compounds were eluted with different gradients of MeCN in H_2_O, at a flow rate of 2.0 mL/min.

### Computer-Assisted Sequence Analysis

The nucleotide and amino acid sequences were downloaded from the National Center for Biotechnology Information (NCBI) database (https://www.ncbi.nlm.nih.gov). Genomic DNA and the gene cluster were analyzed with antiSMASH 6.0 (https://antismash.secondarymetabolites.org/#!/start). The BLAST tool from NCBI (https://blast.ncbi.nlm.nih.gov/Blast.cgi) and the SnapGene software version 8.0.2 (www.snapgene.com) were used for nucleotide and protein sequence alignments. SnapGene was also used for PCR simulation, plasmid design and analysis of sequencing results.

### Strains, Media, and Growth Conditions

Competent *E. coli* DH5α and ET12567 cells were used for plasmid construction and conjugation into *Streptomyces albus* J1074, respectively. They were cultivated at 37 °C and 230 rpm in liquid lysogeny broth (LB) medium (tryptone 10.0 g/L, yeast extract 5.0 g/L, NaCl 10.0 g/L).

*Kitasatospora azatica* NRRL B-24283 was kindly provided by the ARS Culture Collection (NRRL) and cultivated in liquid GYM *Streptomyces* medium (glucose 4.0 g/L, yeast extract 4.0 g/L, malt extract 10.0 g/L, CaCO_3_ 2.0 g/L, pH 7.2) at 28 °C and 180 rpm to get extract for metabolite detection and to obtain biomass for subsequent genomic DNA isolation as described previously.^25^ For sporulation, *K. azatica* was cultivated on GYM *Streptomyces* medium with agar (20 g/L).

Sporulation of *S. albus* J1074 and the corresponding exconjugants was induced on solid mannitol-soy (MS) medium (mannitol 20 g/L, soy flour 20 g/L, agar 20 g/L) supplemented with apramycin (50 μg/mL), if necessary. For secondary metabolite production, the strains were cultivated in modified R5 liquid medium (sucrose 103 g/L, glucose 10 g/L, tryptone 0.1 g/L, yeast extract 5 g/L, K_2_SO_4_ 0.25 g/L, MgCl_2_·6 H_2_O 12 g/L, MOPS 21 g/L, trace elements 2 mL/L, pH 7.2) and incubated at 28 °C and 180 rpm. A complete list of all strains used in this study can be found in Table S2.

### Gene Amplification, Cloning and Plasmid Construction

All genes used in this study were amplified from the genomic DNA of *K. azatica* NRRL B-24283 via PCR by using the Q5 High-Fidelity DNA Polymerase (New England Biolabs GmbH, Frankfurt am Main, Germany) according to the manufacturer’s protocols. The primers used in this study were synthesized by SeqLab GmbH (Göttingen, Germany). The obtained PCR fragments were inserted into a linearized pPWW50A vector by homologous recombination and cloned into competent *E. coli* DH5α.^23^ The nucleotide sequences of the PCR fragments were confirmed by sequencing (SeqLab GmbH). The plasmids were afterward used for heterologous gene expression in *S. albus* J1074. A list of all primers and plasmids used in this study can be found in Table S3.

### Heterologous Gene Expression in *S. albus* J1074

Both expression plasmids pPhM02 and pPhM03 were transformed into competent *E. coli* ET12567 cells and conjugated into *S. albus* J1074 on solid MS agar medium supplemented with MgCl_2_·6 H_2_O (20 mM) as described previously.^26^ The resulting exconjugants were selected and sporulated on MS agar plates containing apramycin (50 μg/mL).

### Fermentation, Extraction, and Isolation

The pPhM02 and pPhM03 harboring *S. albus* strains were cultivated in 8 × 2-L-unbaffled Erlenmeyer flasks, each containing 500 mL of modified R5 medium, at 28 °C and 180 rpm for 7 days. The supernatant was separated from the cell pellet by centrifugation using a Thermo Scientific Fresco 17 microcentrifuge (Thermo Scientific, Dreieich, Germany). One liter of the resulting culture supernatant was extracted twice with 800 mL of EtOAc. The combined EtOAc phases were evaporated to dryness, and the crude extract was used for further purification. For isolation, the EtOAc extracts of *S. albus* exconjugants were fractionated by silica gel 60 (0.04–0.063 mm and 0.2–0.5 mm) column chromatography, eluted with a gradient of CH_2_Cl_2_ and MeOH from 100:10 to 0:100 (v/v), which were then purified on a semipreparative HPLC with an Agilent HPLC series 1200 (Agilent Technologies) equipped with a VDSpher PUR 100 C18-M-SE column (250 × 10 mm, 5 μm) (VDS Optilab Chromatographie Technik) using gradients of ACN in H_2_O, at a flow rate of 2.0 mL/min.

### LC-MS Analysis

Natural product analysis was performed using an Agilent 1260 HPLC system (Agilent Technologies) connected to a micrOTOF-Q III mass spectrometer (Bruker, Bremen, Germany). Chromatographic separation was achieved on a VDSpher PUR 100 C18-M-SE column (150 × 2 mm, 3 μm; VDS Optilab Chromatographietechnik GmbH). The separation was carried out using a linear gradient of 5–100% MeCN in H_2_O, both containing 0.1% HCOOH, in 10 min and a flow rate at 0.3 mL/min was used. Subsequently the column was washed with 100% MeCN containing 0.1% HCOOH for 5 min and equilibrated with 5% MeCN in H_2_O for 5 min.

### Physicochemical Properties of the Compounds Described in This Study

*Cyclo*-l-Trp-l-Trp (**1**): White powder; HRESIMS (Figure S2) *m*/*z*: 373.1659 [M + H]^+^ (calcd. for C_22_H_20_N_4_O_2_: 373.1659); ^1^H NMR data are listed in Table S4 and ^1^H NMR spectrum as Figure S5.

Guanitrypmycin D3 (**2a**): Pale yellow powder; HRESIMS (Figure S2) *m*/*z*: 522.2054 [M + H]^+^ (calcd. for C_27_H_24_N_9_O_3_: 522.1997); NMR data are listed in Table S5 and NMR spectra as Figures S6–11.

Uncharacterized guanitrypmycin (**2b**): HRESIMS (Figure S2) *m*/*z*: 522.2026 [M + H]^+^ (calcd. for C_27_H_24_N_9_O_3_: 522.1997).

Guanitrypmycin C3–3 (**3a**): Pale yellow powder; HRESIMS (Figure S2) *m*/*z*: 507.1923 [M + H]^+^ (calcd. for C_27_H_23_N_8_O_3_: 507.1888); ^1^H NMR data are listed in Table S4 and ^1^H NMR spectrum as Figure S12.

Guanitrypmycin C3–5 (**3b**): Pale yellow powder; HRESIMS (Figure S2) *m*/*z*: 507.1920 [M + H]^+^ (calcd. for C_27_H_23_N_8_O_3_: 507.1888); NMR data are listed in Table S5 and NMR spectra as Figures S13–18.

## Supplementary Material





## Notes

Primers; plasmids; strains; MS; NMR tables; spectra (PDF)
Raw 1D and 2D NMR data for guanitrypmycin C3–5 (**3b**) (ZIP)
