# Supplementary material for: Elucidation of the Guanitrypmycin Biosynthetic Pathway in Kitasatospora azatica Leading to Structure Revision and Reassignment
Source: J Nat Prod. 2026 Apr 14;89(4):1355–61. doi: 10.1021/acs.jnatprod.6c00242 (PMC13122648; doi:10.1021/acs.jnatprod.6c00242)
Supplement: Supplementary file 1 [file np6c00242_si_001.pdf]

Supporting Information

Elucidation of the Guanitrypmycin Biosynthetic Pathway in *Kitasatospora azatica* Leading to Structure Revision and Reassignment

Philipp Mann and Shu-Ming Li*

Philipps-Universität Marburg, Fachbereich Pharmazie, Institut für Pharmazeutische Biologie und Biotechnologie, Robert-Koch-Straße 4, Marburg 35037, Germany.

*Email: shuming.li@staff.uni-marburg.de

Table of contents

Supplementary Tables 3

Table S1. Putative functions of the *gut*$_{24283}$ gene cluster 3

Table S2. Bacterial strains used in this study. 3

Table S3. Primers, cloning sites and plasmids used in this study 4

Table S4. ^{1}H NMR data of cWW (**1**) and guanitrypmycin C3-3 (**3a**) 5

Table S5. NMR data of guanitrypmycin D3 (**2a**) and guanitrypmycin C3-5 (**3b**) 6

Supplementary Figures 7

Figure S1. LC-MS chromatograms of the culture extract of *K. azatica* 7

Figure S2. MS spectra of the five *gut*$_{24283}$ products 8

Figure S3. Comparison of ^{13}C NMR spectra of **2a** and relevant guanitrypmycins 9

Figure S4. Comparison of ^{13}C NMR spectra of **3b** and guanitrypmycin C3-4 9

Figure S5. ^{1}H NMR spectrum of **1** in DMSO-*d6* (500 MHz) 10

Figure S6. ^{1}H NMR spectrum of **2a** in DMSO-*d6* (500 MHz) 10

Figure S7. ^{13}C NMR spectrum of **2a** in DMSO-*d6* (125 MHz) 11

Figure S8. ^{1}H-^{1}H COSY spectrum of **2a** in DMSO-*d6* 11

Figure S9. HSQC spectrum of **2a** in DMSO-*d6* 12

Figure S10. HMBC spectrum of **2a** in DMSO-*d6* 12

Figure S11. NOESY spectrum of **2a** in DMSO-*d6* 13

Figure S12. ^{1}H NMR spectrum of **3a** in DMSO-*d6* (500 MHz) 13

Figure S13. ^{1}H NMR spectrum of **3b** in DMSO-*d6* (500 MHz) 14

Figure S14. ^{13}C NMR spectrum of **3b** in DMSO-*d6* (125 MHz) 14

Figure S15. ^{1}H-^{1}H COSY spectrum of **3b** in DMSO-*d6* 15

Figure S16. HSQC spectrum of **3b** in DMSO-*d6* 15

Figure S17. HMBC spectrum of **3b** in DMSO-*d6* 16

Figure S18. NOESY spectrum of **3b** in DMSO-*d6* 16

References 17

SUPPLEMENTARY TABLES

Table S1. Putative functions of the *gut*$_{24283}$ gene cluster

Protein (Accession no.)	Size (aa)	Protein (Accession no.)	Sequence identity (%)	Protein (Accession no.)	Sequence identity (%)	Putative function
GutA$_{24283}$ (WP_083976074)	247	PtmA (WP_035796170)	72.6	GutA$_{2774}$ (WP_078950527)	50.2	tRNA-dependent cyclodipeptide synthase
GutD$_{24283}$ (WP_035841180)	398	PtmB (WP_051966480)	86.7	GutD$_{2774}$ (WP_051841251)	66.3	cytochrome p450

Table S2. Bacterial strains used in this study

Strain	Source	Cultivation medium
Escherichia coli DH5α	Thermo Fisher Scientific. Inc. (Waltham, MA, USA)	LB
Escherichia coli ET12567/pUZ8002	[1]	LB
Kitasatospora azatica NRRL B-24283	USDA Agricultural Research Service (ARS): Culture Collection (NRRL)	GYM streptomyces medium
Streptomyces albus J1074	[2]	MS and modified R5 medium

LB medium: tryptone 10.0 g/L, yeast extract 5.0 g/L, NaCl 10.0 g/L

GYM streptomyces medium: glucose 4.0 g/L, yeast extract 4.0 g/L, malt extract 10.0 g/L, $CaCO_3$ 2.0 g/L, pH 7.2

MS medium: mannitol 20 g/L, soy flour 20 g/L, agar 15 g/L

Modified R5 medium: sucrose 103 g/L, glucose 10 g/L, tryptone/peptone 0.1 g/L, yeast extract 5 g/L, K_2SO_4 0.25 g/L, $MgCl_2 \cdot 6\ H_2O$ 12 g/L, MOPS 21 g/L, trace elements 2 mL/L, pH 7.2

Trace element solution: $ZnCl_2$ 40 mg/L, $FeCl_3 \cdot 6\ H_2O$ 200 mg/L, $CuCl_2 \cdot 2\ H_2O$ 10 mg/L, $MnCl_2 \cdot 4\ H_2O$ 10 mg/L, $Na_2B_4O_7 \cdot 10\ H_2O$ 10 mg/L, $(NH_4)_6Mo_7O_{24} \cdot 4\ H_2O$ 810 mg/L

Table S3. Primers, cloning sites and plasmids used in this study

Gene(s)	Primer sequences (5'-3')	Cloning site	Expression vector	Gene position and orientation in the construct	Plasmid
gutA$_{24283}$	cacagcagcggccatatcgaaggtcgtcatatgagcaattcgctcctcagcaccaag<br>atgcagagcttctagaactagtggatcctctcaggccgcggcgctgag	NdeI/ BamHI	pPWW50A	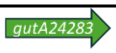	pPhM02
gutAD$_{24283}$	cacagcagcggccatatcgaaggtcgtcatatgagcaattcgctcctcagcaccaag<br>atgcagagcttctagaactagtggatcctcctaccagatcatcgggaggcgctcc	NdeI/ BamHI	pPWW50A	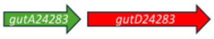	pPhM03

Nucleotides overlapping with the vector backbone are underlined.

Table S4. ^{1}H NMR data of cWW (**1**) and Guanitrypmycin C3-3 (**3a**) in DMSO-d_6 (500 MHz)

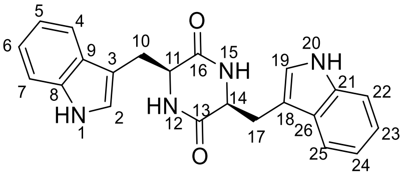

1

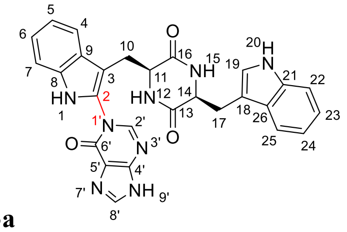

3a

	**1**	**3a**
	δ_H, multi. (J in Hz)	δ_H, multi. (J in Hz)
NH-1	10.81, s	11.56, s
2	6.59, br s	—
4	7.33, d (8.0)	7.20, d (7.5) [a]
5	6.93, br t (8.0)	7.04, td (7.5, 1.0)
6	7.02, br t (8.0)	7.19, td (7.5, 1.1) [a]
7	7.27, d (8.0)	7.35, d (7.5)
10	2.69, dd (14.3, 4.1)<br>2.18, dd (14.3, 6.7)	2.74, dd (15.0, 4.4)<br>2.36, m [b]
11	3.85, m	3.82, m
NH-12	7.65, br s	7.59, d (2.6)
14	3.85, m	3.89, m
NH-15	7.65, br s	7.85, d (2.6)
17	2.69, dd (14.3, 4.1)<br>2.18, dd (14.3, 6.7)	2.77, dd (15.2, 4.4)<br>2.34, m [b]
19	6.59, br s	6.62, d (1.9)
NH-20	10.81, s	10.76, d (1.9)
22	7.27, d (8.0)	7.27, d (8.1)
23	7.02, br t (8.0)	6.99, td (8.1, 1.0)
24	6.93, br t (7.5)	6.90, td (7.6, 1.0)
25	7.33, d (8.0)	7.34, d (7.6)
2'	—	8.15, s
8'	—	8.21, br s
NH-9'	—	Not obs.

[a, b]: signals with the same letter overlapping with each other

The data of **1** and **3a** correspond to those of cWW and guanitrypmycin C3-3, respectively.[3,4]

Table S5. NMR data of guanitrypmycin D3 (**2a**) and guanitrypmycin C3-5 (**3b**) in DMSO-d_6

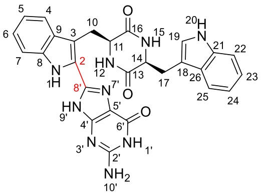

2a

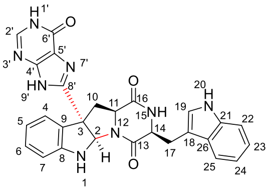

3b

	δ_C, type	δ_H, multi. (J in Hz)	δ_C, type	δ_H, multi. (J in Hz)
NH-1	—	7.63, br s	—	6.99, br s
2	123.9, C	—	81.4, CH	6.32, br s
3	114.1, C	—	56.4, C	—
4	119.2, CH	7.30, m [a]	122.6, CH	6.97, m [b]
5	121.4, CH	7.16, t (7.3)	117.6, CH	6.57, br t (7.4) [c]
6	123.3, CH	7.29, m [a]	128.7, CH	7.04, br t (7.4) [d]
7	114.1, CH	8.42, d (8.1)	109.5, CH	6.60, br d (7.6) [c]
8	134.6, C	—	148.4, C	—
9	129.2, C	—	130.1, C	—
10	29.7, CH_2	2.71, dd (14.6, 3.7)<br>2.11, dd (14.6, 7.3)	39.2, CH_2 [x]	3.37, t (4.8) [e, x]<br>2.08, dd (11.3, 12.7)
11	55.0, CH	3.95, dt (7.3, 3.7)	58.1, CH	4.45, dd (11.3, 6.4)
NH-12	—	7.89, d (2.6)	—	—
13	166.7, C	—	167.5, C	—
14	55.5, CH	3.99, t (6.7)	55.1, CH	4.41, t (5.7)
NH-15	—	7.91, d (2.6)	—	7.71, s
16	166.4, C	—	169.4, C	—
17	29.9, CH_2	2.82, dd (14.3, 4.3)<br>2.56, dd (14.3, 6.7) [x]	24.5, CH_2	3.37, m [e, x]<br>3.03, dd (15.4, 6.7)
18	108.7, C	—	109.4, C	—
19	124.2, CH	6.82, d (2.4)	124.1, CH	7.25, d (2.4)
NH-20	—	10.85, d (2.4)	—	10.83, d (2.4)
21	136.0, C	—	136.0, C	—
22	111.3, CH	7.30, m [a]	111.2, CH	7.32, d (8.1)
23	120.9, CH	7.02, t (7.3)	120.9, CH	7.06, br t (8.1) [d]
24	118.4, CH	6.91, t (7.3)	118.2, CH	6.97, m [b]
25	118.5, CH	7.38, d (8.0)	118.4, CH	7.57, d (7.4)
26	127.5, C	—	127.3, C	—
NH-1'	—	10.63, br s	—	Not obs.
2'	Not obs.	—	Not obs.	7.96, s
4'	148.8, C	—	Not obs.	—
5'	Not obs.	—	Not obs.	—
6'	153.3, C	—	Not obs.	—
8'	138.8, C	—	Not obs.	—
NH-9'	—	Not obs.	—	12.17, br s
NH_2-10'	Not obs.	6.28, br s	—	—

[a, b, c, d, e]: signals overlapping with each other; [x]: signals overlapping with the solvent peak.

The NMR data of **2a** correspond well to those of guanitrypmycin D3 and of the misassigned guanitrypmycin C3-1.[5] The NMR data of **3b** correspond to those of the incorrectly revised structure of guanitrypmycin C3-4 published previously.[6]

SUPPLEMENTARY FIGURES

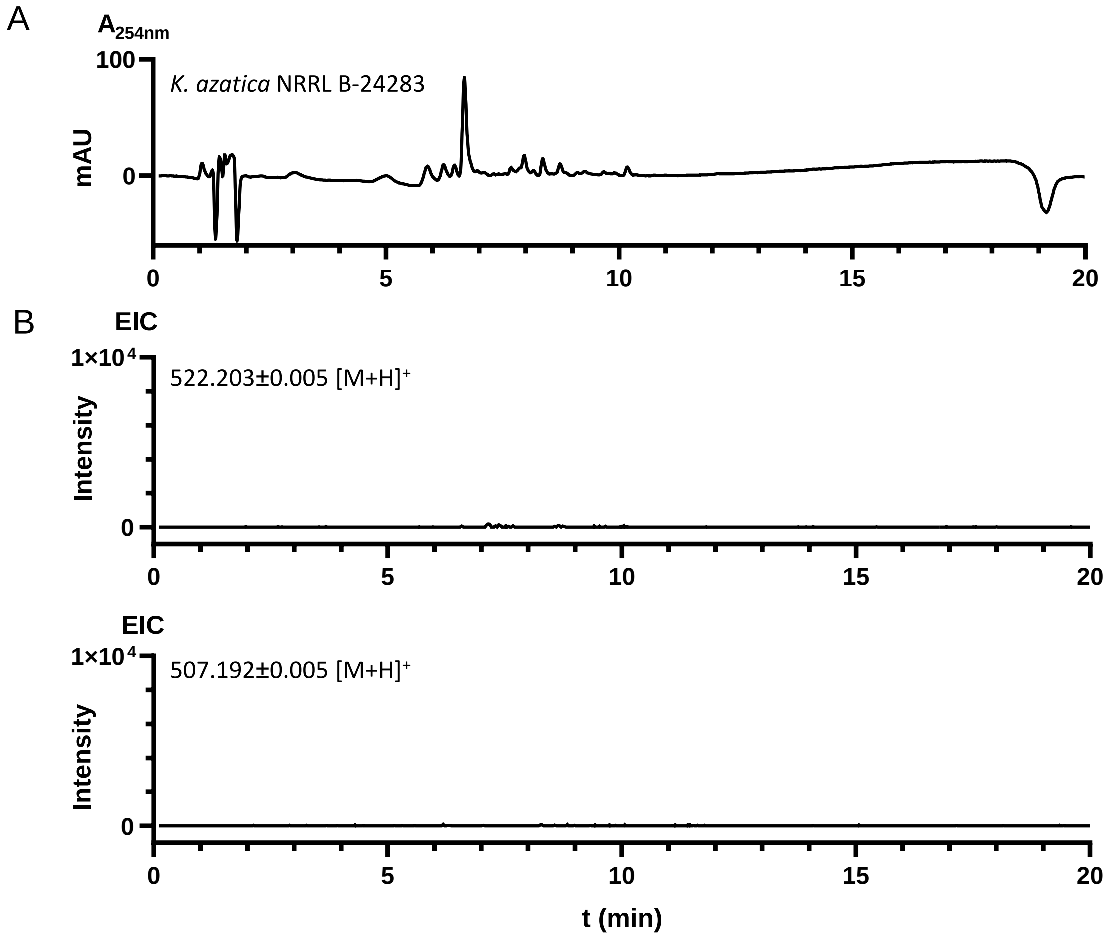

Figure S1. LC-MS chromatograms of the culture extract of *K. azatica* after 7d cultivation in GYM streptomyces media. A: UV absorptions for 254 nm are illustrated. B: Extracted ion chromatograms (EICs) of $[M+H]^+$ ions of the relevant guanitrypmycins: 522.203±0.005 for cWW with guanine and 507.192±0.005 with hypoxanthine.

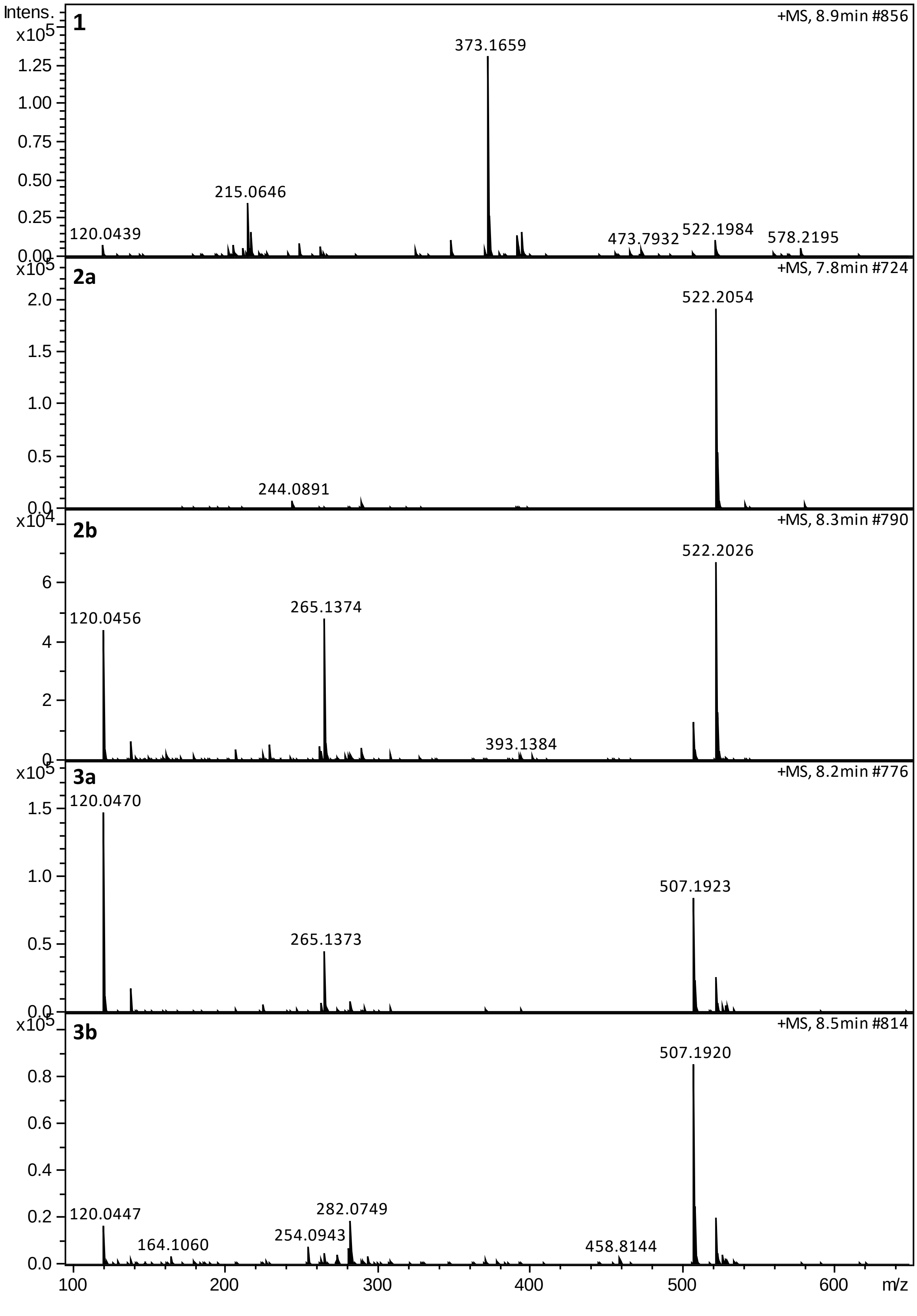

Figure S2. MS spectra of the five *gut*$_{24283}$ products. *cyclo*-L-Trp-L-Trp (**1**), guanitrypmycin D3 (**2a**), not isolated product (**2b**), guanitrypmycin C3-3 (**3a**), and guanitrypmycin C3-5 (**3b**).

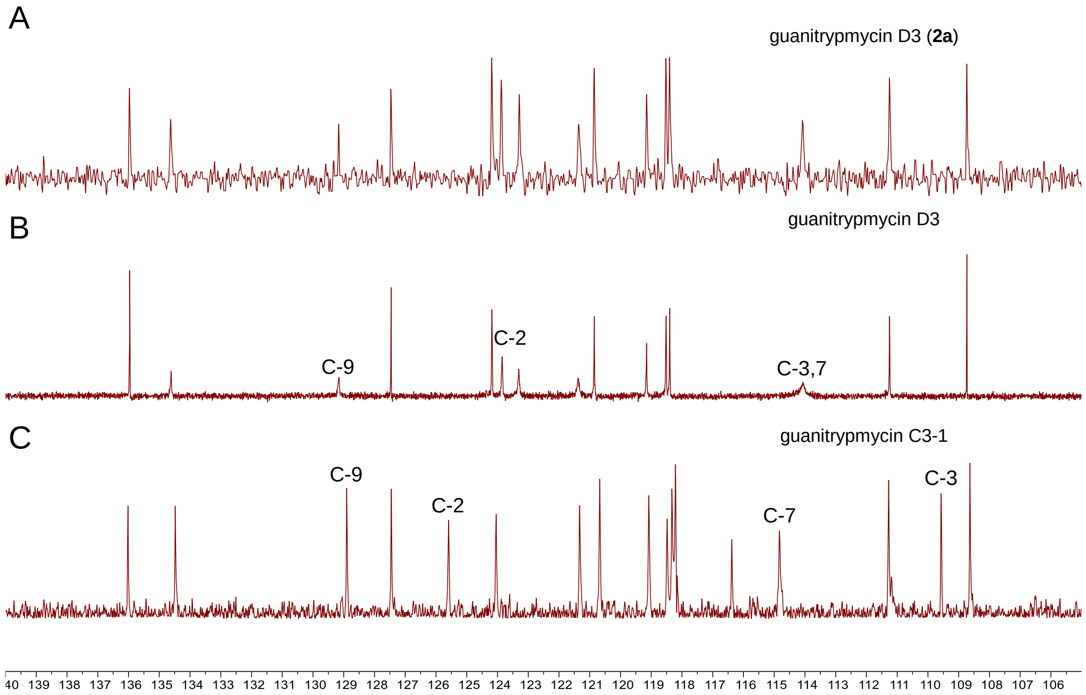

Figure S3. Comparison of ^{13}C NMR spectrum of **2a** (DMSO-*d6*) with those of guanitrypmycin C3-1 and D3 in the range of 105–140 ppm. Characteristic signals are labelled. (A) Spectrum of compound **2a** isolated in this study, identified as guanitrypmycin D3. (B) Spectrum of guanitrypmycin D3. The spectrum was generated using raw data originally reported by our group.[5] (C) Spectrum of guanitrypmycin C3-1. The spectrum was generated using raw data originally reported by our group.[7]

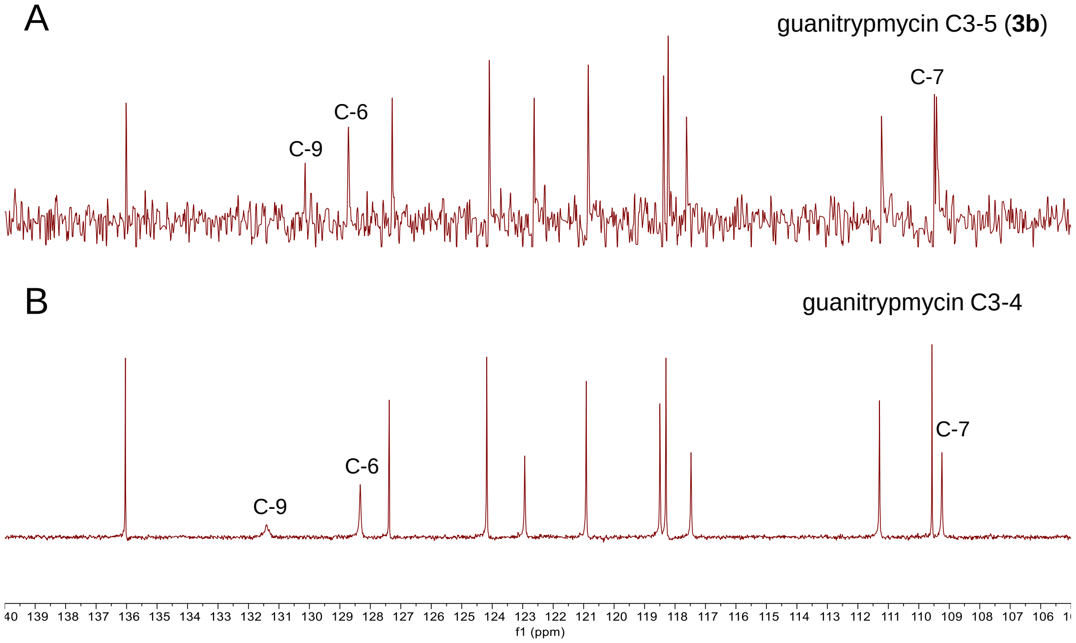

Figure S4. Comparison of ^{13}C NMR spectrum of **3b** (DMSO-*d6*) with that of guanitrypmycin C3-4 in the range of 105–140 ppm. Characteristic signals are labelled. (A) Spectrum of compound **3b** isolated in this study, identified as guanitrypmycin C3-5. (B) Spectrum of guanitrypmycin C3-4. The spectrum was generated using raw data originally reported by our group.[4]

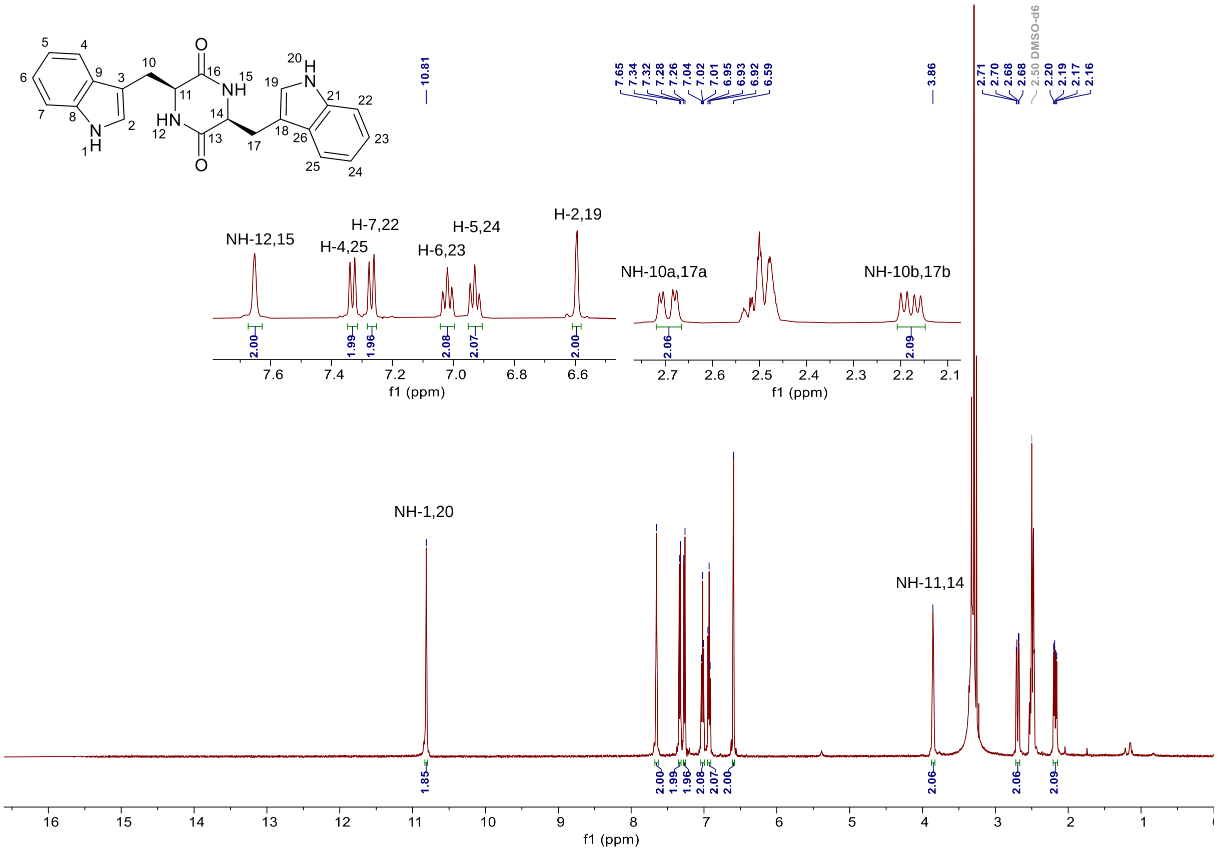

Figure S5. ^{1}H NMR spectrum of **1** in DMSO-*d6* (500 MHz).

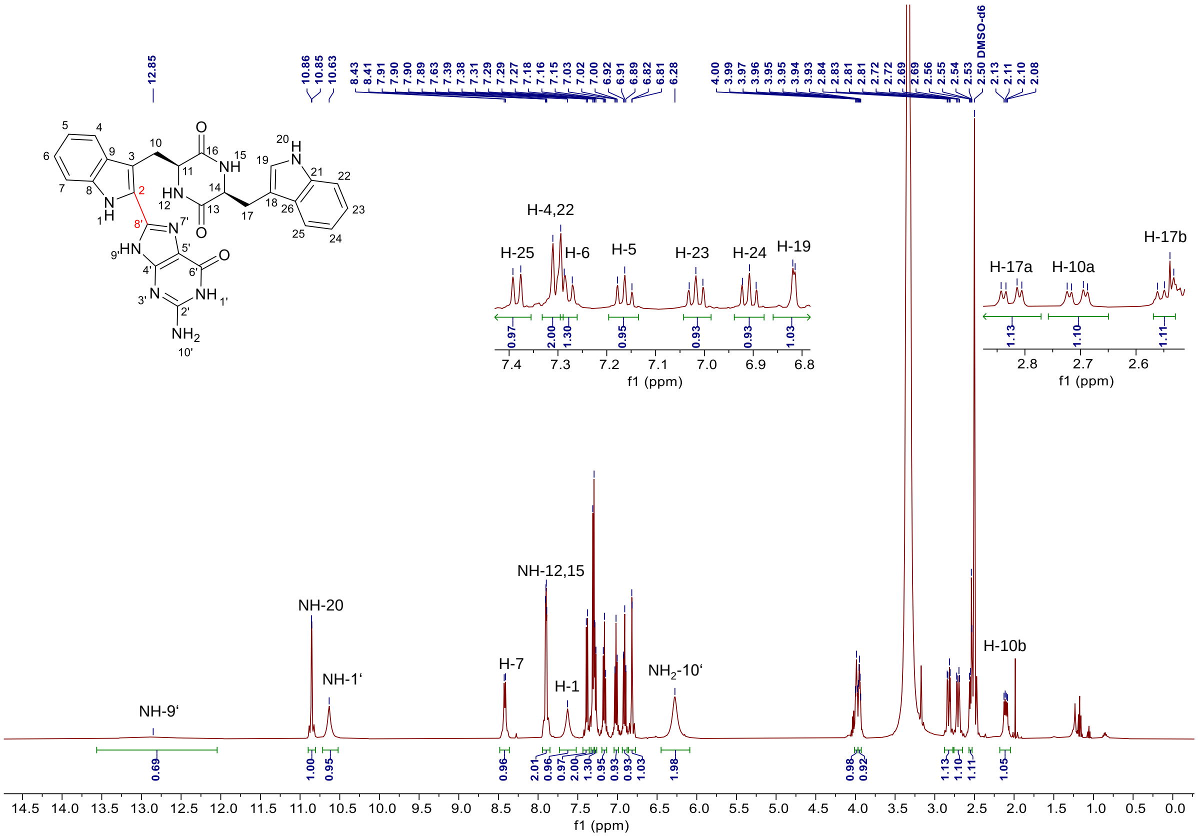

Figure S6. ^{1}H NMR spectrum of **2a** in DMSO-*d6* (500 MHz).

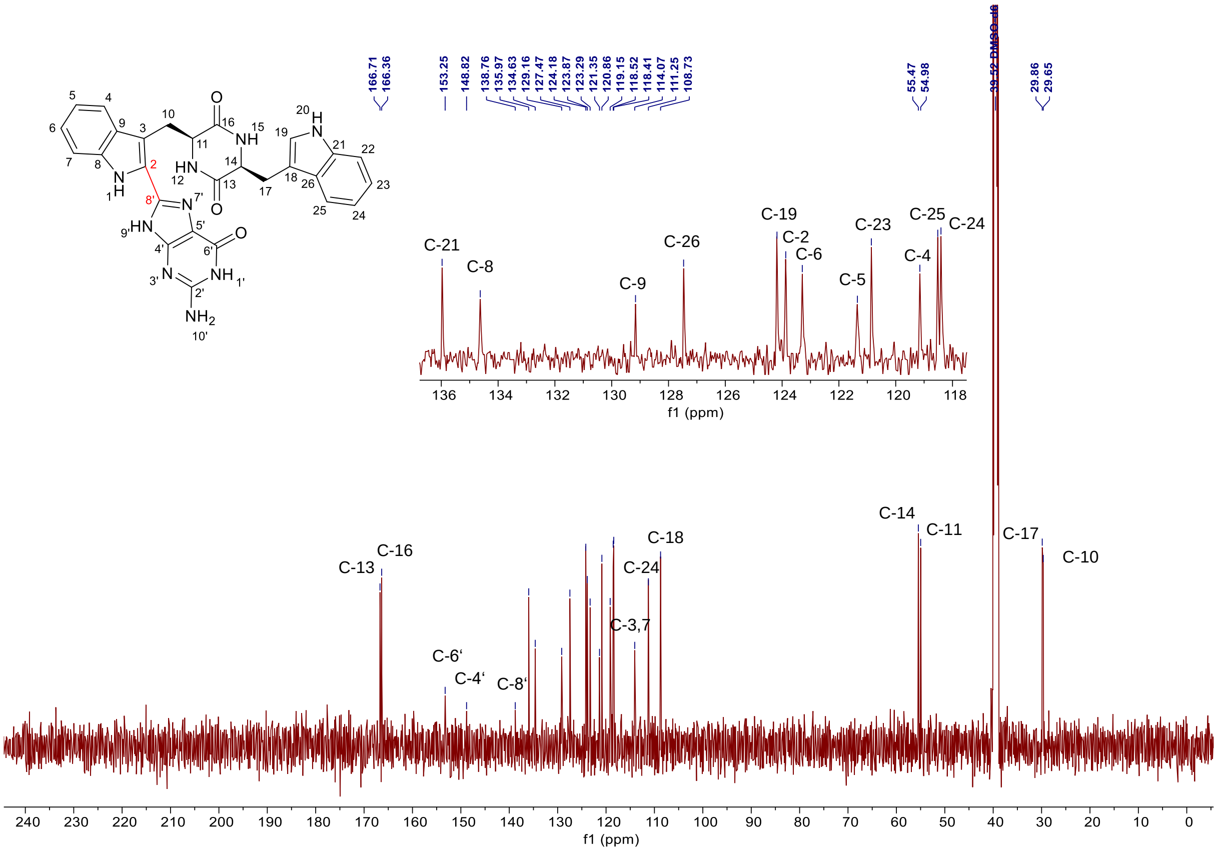

Figure S7. ^{13}C NMR spectrum of **2a** in DMSO-*d6* (125 MHz).

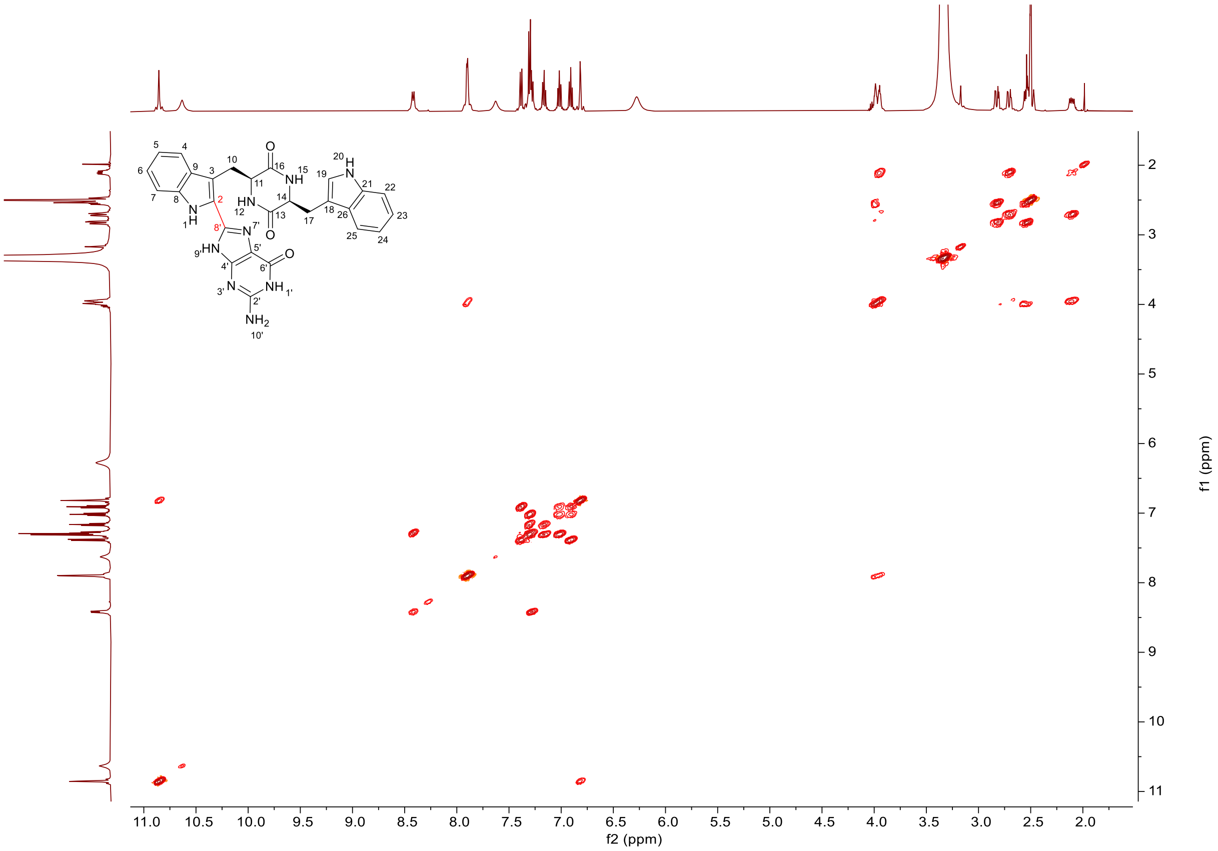

Figure S8. ^{1}H-^{1}H COSY spectrum of **2a** in DMSO-*d6*.

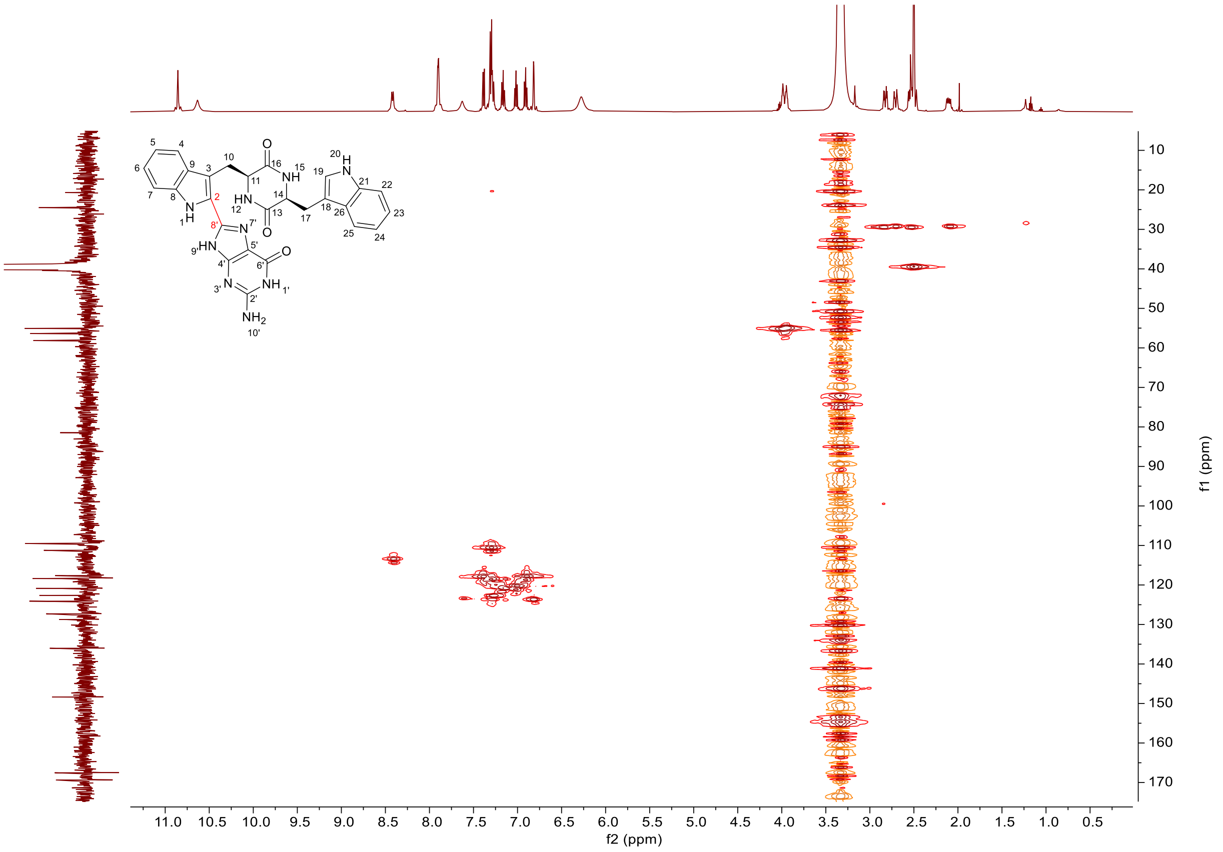

Figure S9. HSQC spectrum of **2a** in DMSO-*d6*.

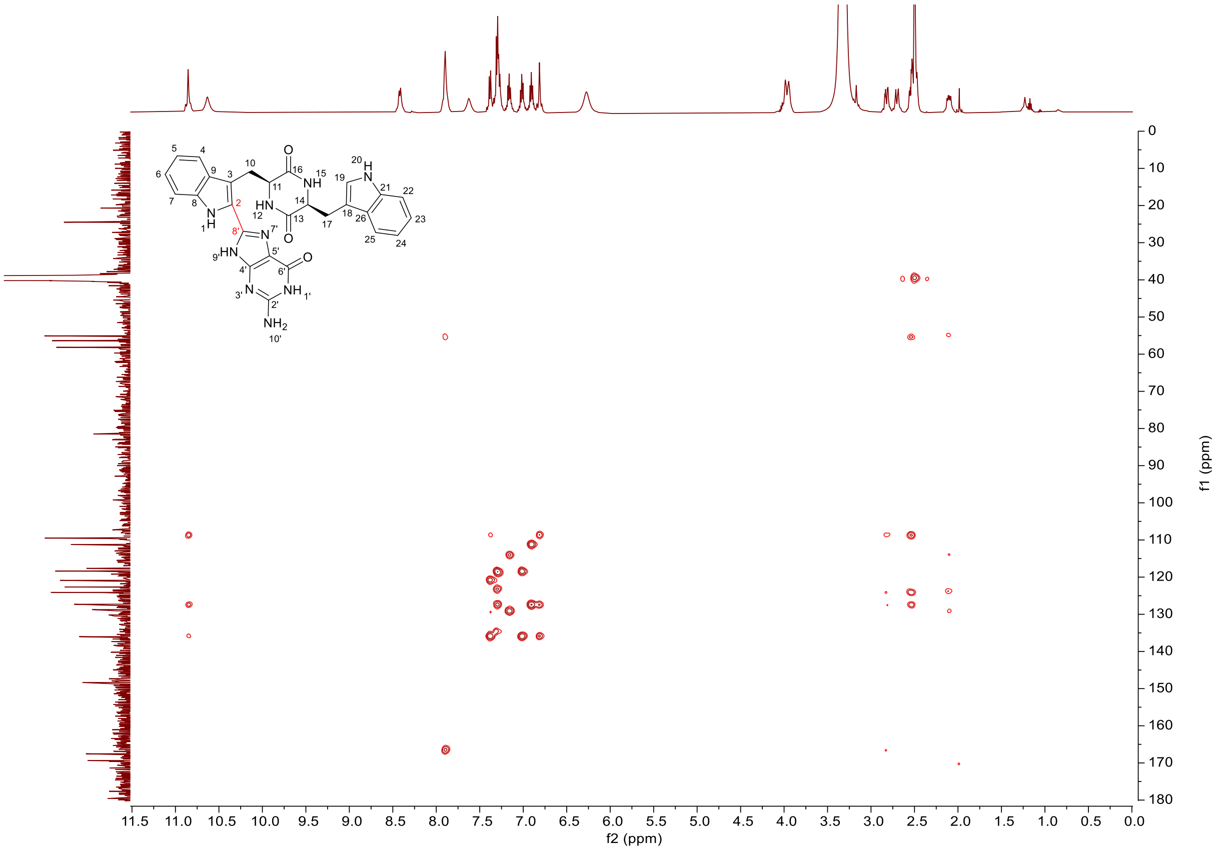

Figure S10. HMBC spectrum of **2a** in DMSO-*d6*.

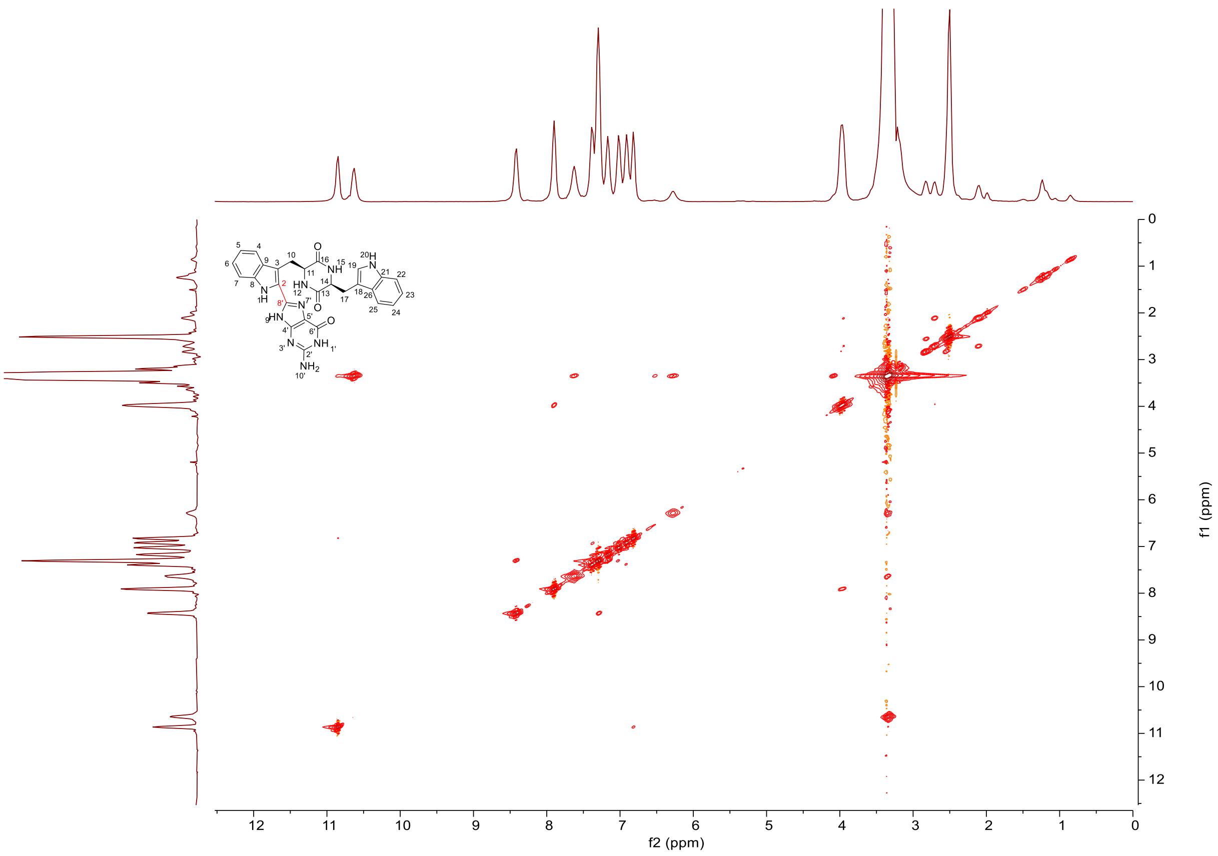

Figure S11. NOESY spectrum of **2a** in DMSO-*d6*.

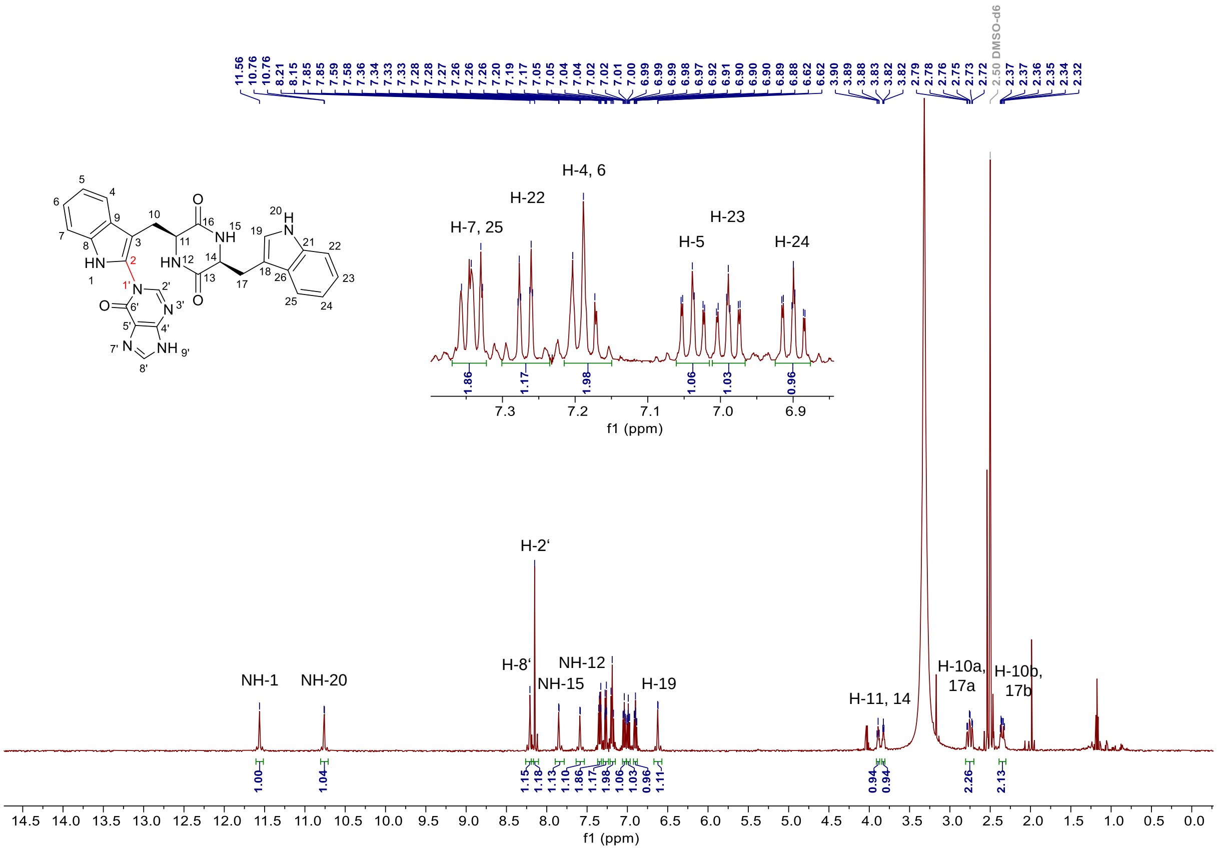

Figure S12. ^{1}H NMR spectrum of **3a** in DMSO-*d6* (500 MHz).

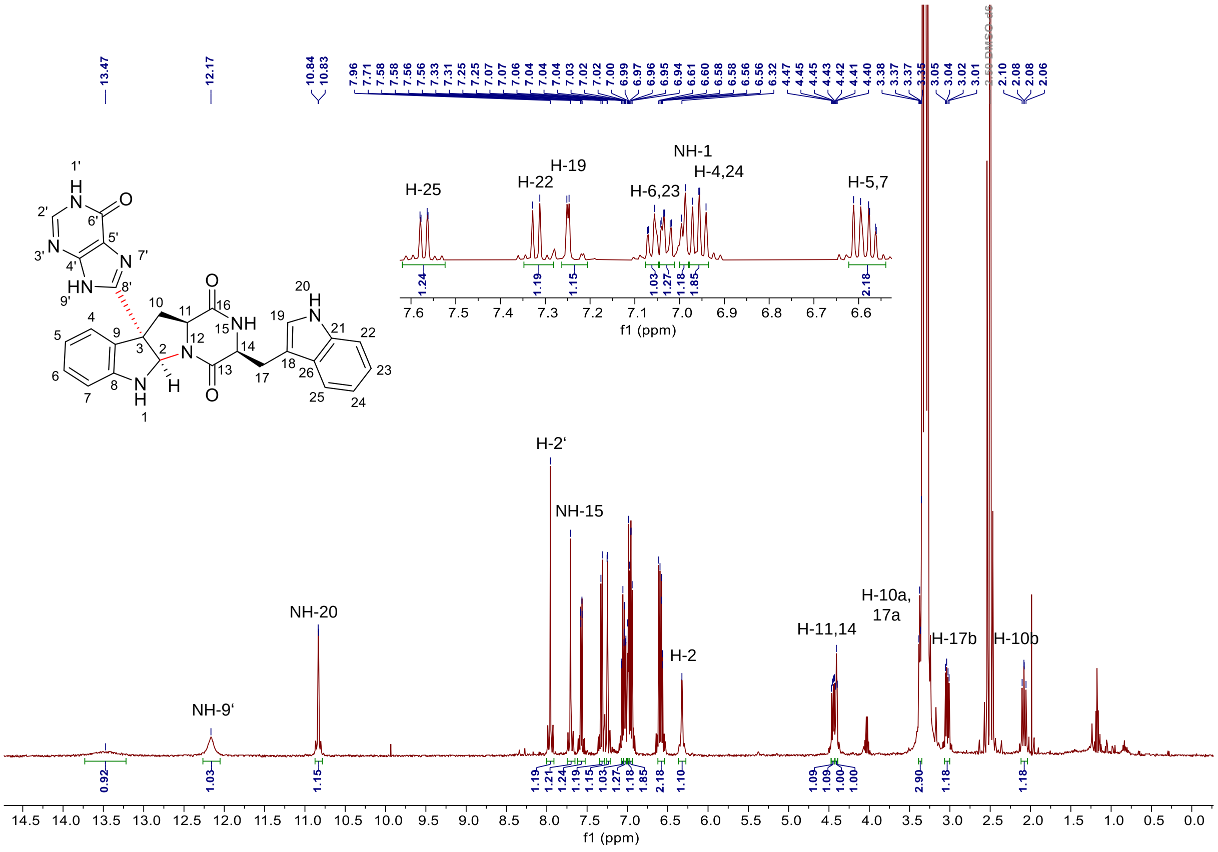

Figure S13. ^{1}H NMR spectrum of **3b** in DMSO-*d6* (500 MHz).

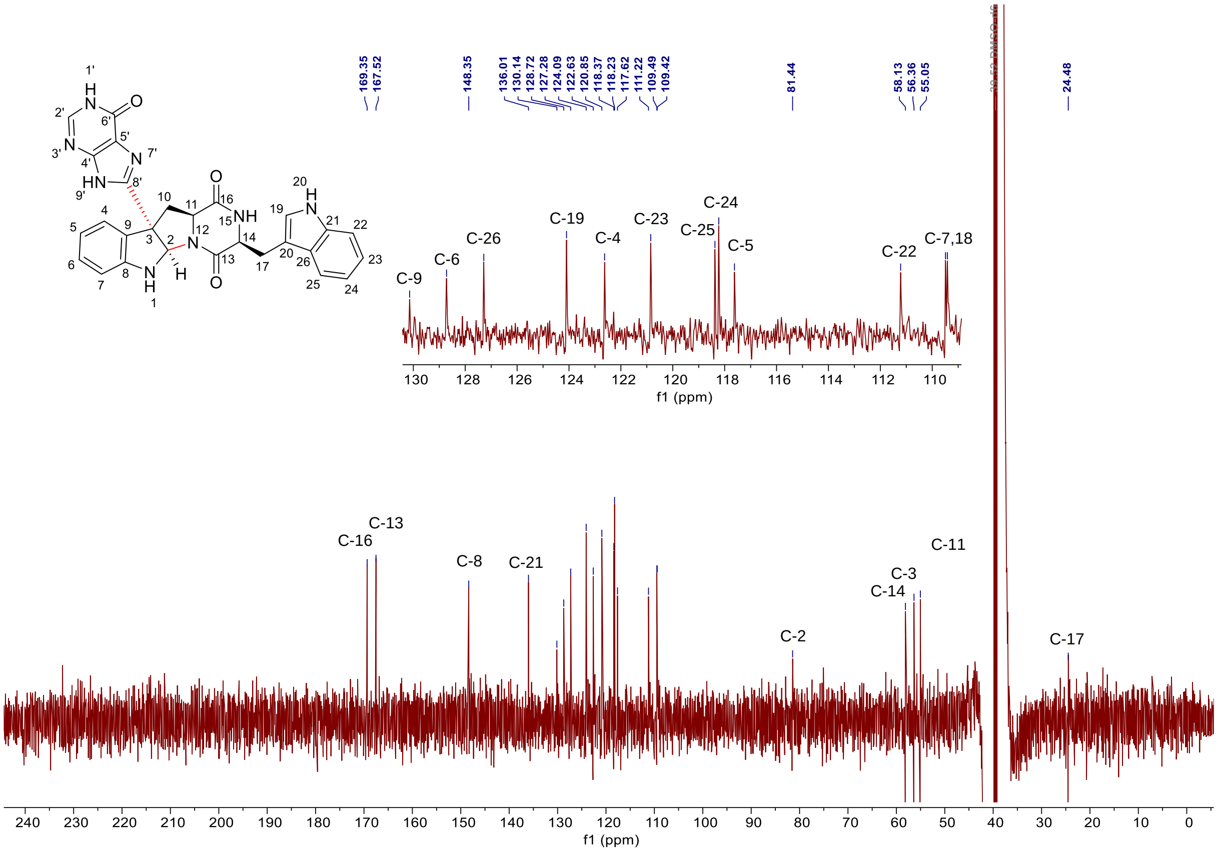

Figure S14. ^{13}C NMR spectrum of **3b** in DMSO-*d6* (125 MHz).

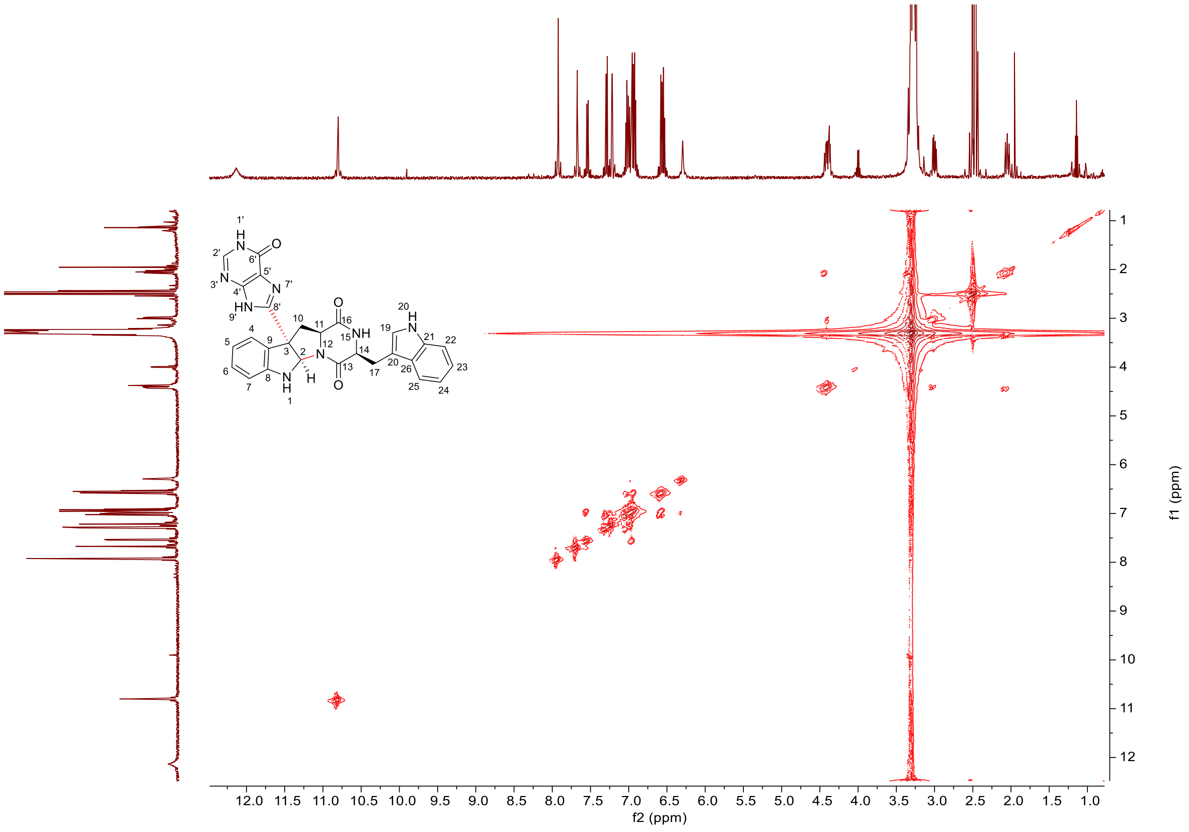

Figure S15. ^{1}H-^{1}H COSY spectrum of **3b** in DMSO-*d6*.

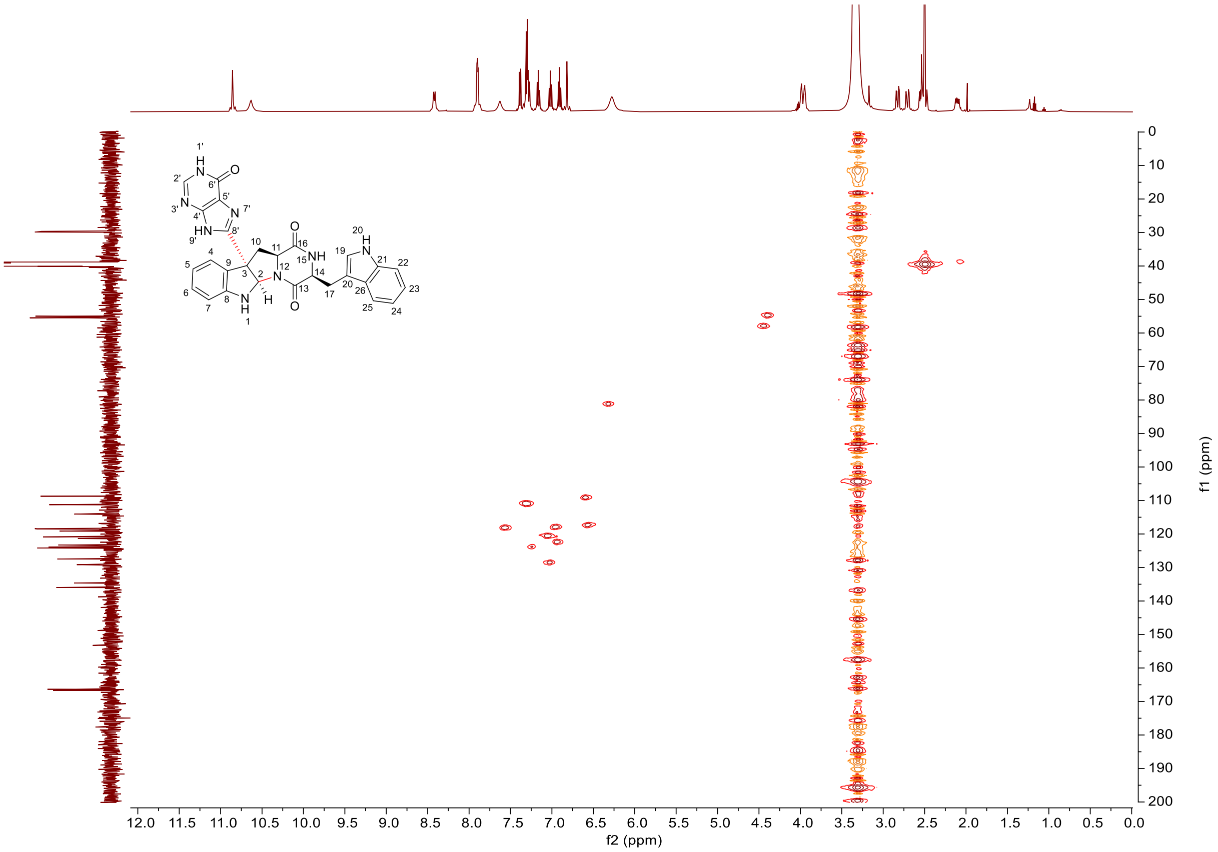

Figure S16. HSQC spectrum of **3b** in DMSO-*d6*.

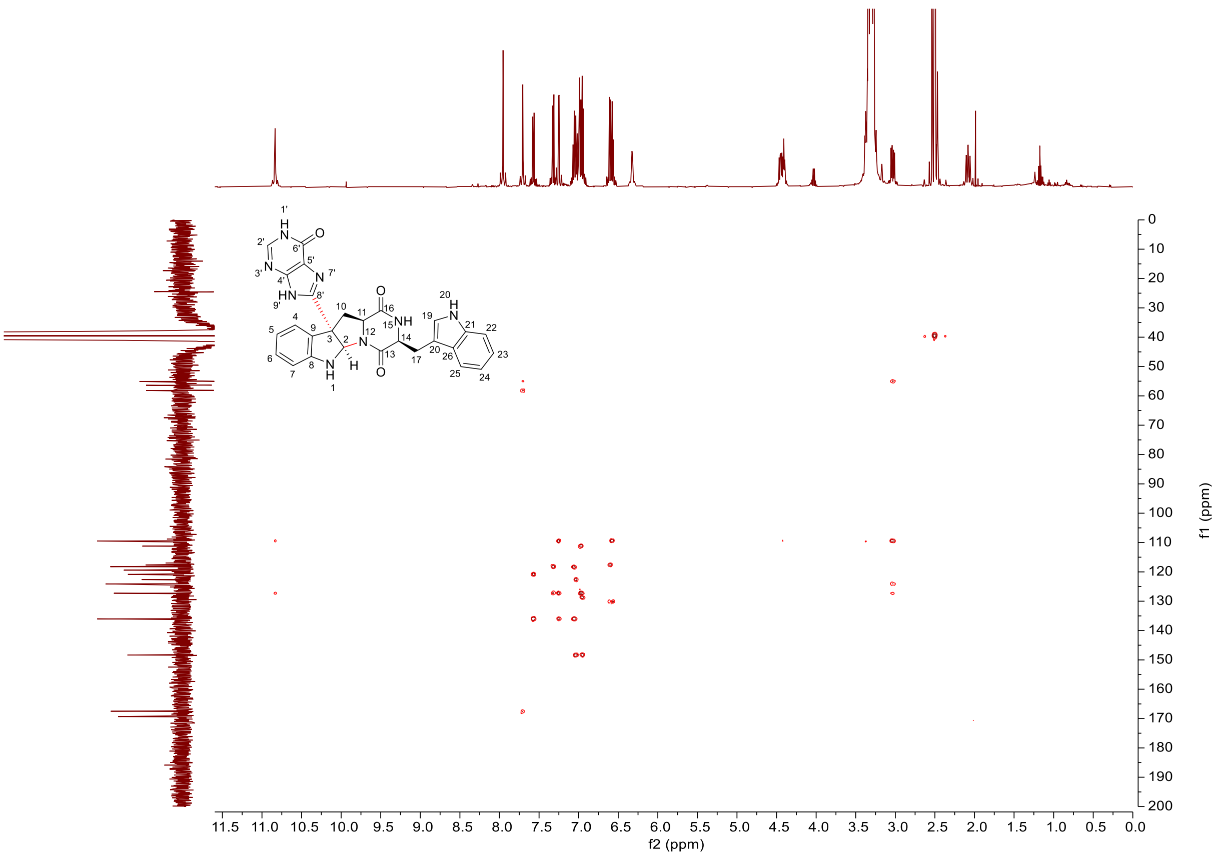

Figure S17. HMBC spectrum of **3b** in DMSO-*d6*.

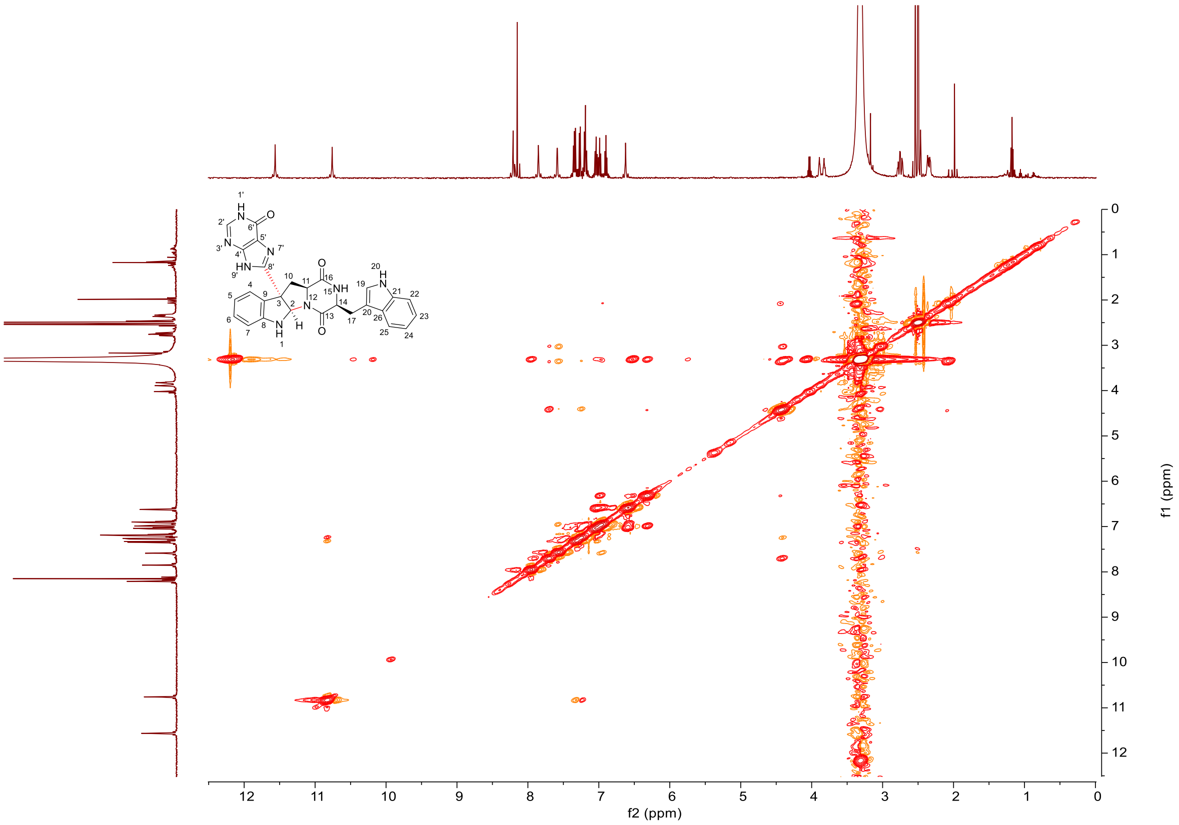

Figure S18. NOESY spectrum of **3b** in DMSO-*d6*.

REFERENCES

(1) Gust, B.; Challis, G. L.; Fowler, K.; Kieser, T.; Chater, K. F. PCR-targeted *Streptomyces* gene replacement identifies a protein domain needed for biosynthesis of the sesquiterpene soil odor geosmin. *Proc. Natl. Acad. Sci. U. S. A.* **2003**, *100* (4), 1541–1546. DOI: 10.1073/pnas.0337542100.

(2) Zaburannyi, N.; Rabyk, M.; Ostash, B.; Fedorenko, V.; Luzhetskyy, A. Insights into naturally minimised *Streptomyces albus* J1074 genome. *BMC Genom.* **2014**, *15*, 97. DOI: 10.1186/1471-2164-15-97.

(3) Liu, J.; Yu, H.; Li, S.-M. Expanding tryptophan-containing cyclodipeptide synthase spectrum by identification of nine members from *Streptomyces* strains. *Appl. Microbiol. Biotechnol.* **2018**, *102* (10), 4435–4444. DOI: 10.1007/s00253-018-8908-6.

(4) Yu, H.; Xie, X.; Li, S.-M. Coupling of *cyclo*-L-Trp-L-Trp with hypoxanthine increases the structure diversity of guanitrypmycins. *Org. Lett.* **2019**, *21*, 9104–9108. DOI: 10.1021/acs.orglett.9b03491.

(5) Liu, J.; Yang, Y.; Xie, X.; Li, S.-M. A *Streptomyces* cytochrome P450 enzyme catalyzes regiospecific C2-guaninylation for the synthesis of diverse guanitrypmycin analogs. *J. Nat. Prod.* **2023**, *86*, 94–102. DOI: 10.1021/acs.jnatprod.2c00787.

(6) Wei, G.; Duan, B.; Zhou, T.-P.; Tian, W.; Sun, C.; Lin, Z.; Deng, Z.; Wang, B.; Zhang, Z.; Qu, X. A nucleobase-driven P450 peroxidase system enables regio- and stereo-specific formation of C─C and C─N bonds. *Proc. Natl. Acad. Sci. U. S. A.* **2024**, *121* (46), e2412890121. DOI: 10.1073/pnas.2412890121.

(7) Yu, H.; Xie, X.; Li, S.-M. Coupling of guanine with *cyclo*-L-Trp-L-Trp mediated by a cytochrome P450 homologue from *Streptomyces purpureus*. *Org. Lett.* **2018**, *20* (16), 4921–4925. DOI: 10.1021/acs.orglett.8b02051.
